# Supplementary material for: Functionalization and higher-order organization of liposomes with DNA nanostructures
Source: Nat Commun. 2023 Aug 29;14:5256. doi: 10.1038/s41467-023-41013-2 (PMC10465589; doi:10.1038/s41467-023-41013-2)
Supplement: Supplementary file 1 — Supplementary information [file 41467_2023_41013_MOESM1_ESM.pdf]

Functionalization and higher-order organization of liposomes with DNA nanostructures

Zhao Zhang, Zhaomeng Feng, Xiaowei Zhao, Dominique Jean, Zhiheng Yu and Edwin R. Chapman

Supplementary Information

TABLE OF CONTENT

Abbreviations 1

Supplementary Materials and Methods 2

- Materials 2
- Protein production 4
- DNA-MSP conjugation 4
- Nanodisc preparation 4
- Liposome preparation 5
- DNA tile design and assembly 5
- DNA origami design and preparation 5
- Liposome coating and clustering by DNA nanostructures 6
- Liposome functionalization and higher-order organization by DNA nanostructures 6
- Liposome 1D array by C-tile assembly 6
- Negative-stain electron microscopy 6
- Cryo-ET 7
- Lipid mixing assay 7

Supplementary Figures 8

- Supplementary Figure 1 8
- Supplementary Figure 2 9
- Supplementary Figure 3 10
- Supplementary Figure 4 11
- Supplementary Figure 5 12
- Supplementary Figure 6 13
- Supplementary Figure 7 14
- Supplementary Figure 8 15
- Supplementary Figure 9 16
- Supplementary Figure 10 17
- Supplementary Figure 11 19

Supplementary Figure 12 20
Supplementary Figure 13 22
Supplementary Figure 14 23
Supplementary Figure 15 24
Supplementary Figure 16 26
Supplementary Figure 17 28
Supplementary Figure 18 29
Supplementary Figure 19 31
Supplementary Figure 20 33
Supplementary Figure 21 34
Supplementary Reference 36

Abbreviations

Abbreviation	Full name
6HB	six helix bundle
BSA	bovine serum albumin
Chol	cholesterol
cryo-ET	cryogenic electron tomography
DDM	n-dodecyl-β-D-maltoside
DND	DNA-tethered nanodisc
DOPC	1,2-dioleoyl-sn-glycero-3-phosphocholine
DOPE	1,2-dioleoyl-sn-glycero-3-phosphoethanolamine
DOPS	1,2-dioleoyl-sn-glycero-3-phospho-L-serine
dsDNA	double-stranded DNA
EDTA	ethylenediaminetetraacetic acid
FRET	fluorescence resonance energy transfer
HEPES	4-(2-hydroxyethyl)-1-piperazineethanesulfonic acid
HPLC	high-performance liquid chromatography
IDT	Integrated DNA Technologies Inc.
MSP	membrane scaffolding protein
NBD-PE	1,2-dioleoyl-sn-glycero-3-phosphoethanolamine-N-(7-nitro-2-1,3-benzoxadiazol-4-yl)
ND	nanodisc
OG	n-octyl-β-D-glucopyranoside
Oligo	oligonucleotide
Rhod-PE	1,2-dioleoyl-sn-glycero-3-phosphoethanolamine-N-(lissamine rhodamine B sulfonyl)
PAGE	polyacrylamide gel electrophoresis
PEG	polyethylene glycol
pf	protein-free
SDS	sodium dodecyl sulfate
SMCC	succinimidyl 4-(N-maleimidomethyl)cyclohexane-1-carboxylate
SNARE	soluble NSF attachment protein receptor
Syb2	synaptobrevin-2
ssDNA	single-stranded DNA
SUV	small unilamellar vesicles
TCEP	(tris(2-carboxyethyl)phosphine)
TEM	transmission electron microscopy
TMSD	toehold-mediated strand displacement

Supplementary Materials and Methods

Materials

Water was purified by an Milli-Q filtration system (Millipore). All oligonucleotides were purchased from Integrated DNA Technologies (IDT) and used without further purification. Cholesterol-modified oligos were HPLC-purified by IDT. Notable oligonucleotides used in this study:

Name	Sequence and modification (5'->3')	Usage
DNA1-5chol	*/5Chol-TEG/*GCCGCAGGATACAGAATACG	most of the studies
DNA2-5biotin	*/5Biosg/*GTGAGTTGTGGTAGATAATTT	link tile and SA; Fig. 1, Supplementary Fig. 5
DNA2-5amine	*/5AmMC6/*GTGAGTTGTGGTAGATAATTT	tether ND; Fig. 1, Supplementary Fig. 7
DNA2-3chol	GTGAGTTGTGGTAGATAATTT*/3CholTEG/*	when ssDNA linker is used; Supplementary Fig. 11 & 19
linker_12	AAATTATCTACCACAACTCACCGTATTCTG TATCCTGCGGC	ssDNA linker for DNA1 & DNA2; Supplementary Fig. 11 & 19
linker_0	GAATCGGTCACAGTACAACCGCGTAGAGT ACGCATAAATAT	dummy linker for DNA1 & DNA2; Supplementary Fig. 19
linker_ds1	GCGGGACTTCTCTAAGCCAGCCGTATTCT GTATCCTGCGGC	dsDNA linker; Supplementary Fig. 11
linker_ds1'	GCTGGCTTAGAGAAGTCCCGCCGTATTCT GTATCCTGCGGC	
DNA1-3chol	GCCGCAGGATACAGAATACG*/3CholTEG/*	zipper conformation; Supplementary Fig. 12
DNA1-5chol-2	*/5Chol-TEG/*<u>TGGAGA</u>GTGAGTTGTGGTAGA TAATTT<u>TGAGGG</u>	when strand displacement is used; Fig. 2c & 5a, Supplementary Fig. 17
DNA1-2'	<u>CCCTCA</u>AAATTATCTACCACAACTCAC<u>TCT CCA</u>	displacing strand; Fig. 2c & 5a, Supplementary Fig. 17
C-chol	TGGACCGATTGATAAGACTTCAGGCCTGA AGTGGTCCACTTATCAATC*TTT/3CholTEG/*	1D liposome array; Fig. 4a, Supplementary Fig. 15
C-tile[1]	TGGACCGATTGATAAGACTTCAGGCCTGA AGTGGTCCACTTATCAATC	ribbon forming; Supplementary Fig. 15

Plasmid used in this study:

Protein	Plasmid	Original source	Reference
MSP_original	MSP1E3D1	Addgene (#20066)	[2]
MSP	MSP1E3D1_E247C	Homemade by mutagenesis	[3]
v-SNARE (synaptobrevin-2)	pTW2	James E. Rothman	[4]
t-SNARE (syntaxin-1A and SNAP-25B)	pTW34	James E. Rothman	[5,6]

Notable chemicals used in this study:

Chemical	Company	Catalog #
DOPC	Avanti Polar Lipids	850375C
DOPE	Avanti Polar Lipids	850725C
DOPS	Avanti Polar Lipids	840035C
Rhod-PE	Avanti Polar Lipids	810150C
NBD-PE	Avanti Polar Lipids	810145C
Glycerol	Thermo Fisher Scientific	BP229-4
SMCC	Thermo Fisher Scientific	22360
SYBR™ Gold	Thermo Fisher Scientific	S11494
Sodium Chloride	Thermo Fisher Scientific	S271
OG	Goldbio	O-110
DDM	Goldbio	DDM
TCEP	Goldbio	TCEP
Chloroform	Sigma-Aldrich	CX1058
HEPES	Sigma-Aldrich	H4034
Magnesium chloride hexahydrate	Sigma-Aldrich	M9272
Potassium chloride	Sigma-Aldrich	P4504
Imidazole	Sigma-Aldrich	56750
Iodixanol	Cosmo Bio USA	AXS-1114542
DNase I	New England Biolabs	M0303S
Uranyl Formate	Electron Microscopy Sciences	16984-59-1
Streptavidin	Millipore	11721666001
SDS	americanBIO	AB01922
Ni Sepharose 6 Fast Flow	Cytiva	17531802
Bio-Beads SM-2 Adsorbents	Bio-Rad	1523920

Notable labware used in this study:

Labware	Company	Catalog #
SW50.1 - Box Of 50 1/2" X 2" Polyclear™ Open Top Ultracentrifuge Tubes	BioComp	151-513
Amicon Ultra-0.5 Centrifugal Filter Unit 30kDa	Millipore	UFC503096
Dialysis tubing (cellulose), flat width 23 mm (0.9 in.), MWCO 14000	Sigma-Aldrich	D0405
Formvar/Carbon 400 mesh, Copper approx. grid hole size: 42μm	Ted Pella	01754-F
96-well Solid White Flat Bottom Polystyrene TC-treated Microplates	Corning	3917

Lipid compositions used in this study:

Lipids	pf-liposome and t-SNARE liposome and pf2 and ND	v-SNARE liposome and pf1
DOPC	65%	62%
DOPE	15%	15%
DOPS	20%	20%
Rhod-PE		1.5%
NBD-PE		1.5%

Protein production

We used established methods to express and purify proteins (MSP, v-SNARE, and t-SNARE) [3,5]. Briefly, *E. coli* strain BL21-Gold (DE3) (Agilent) cells were grown in 2 liters of LB medium in a shaking incubator at 37 °C until the OD reached 0.6, then protein expression was induced with 0.5 mM IPTG for 4 hours at 37 °C. Cultures were then transferred to 2-liter bottles for centrifugation (7,000 × *g* for 15 min). Cell pellets were resuspended in 31 ml of *lysis buffer* (25 mM HEPES, 500 mM NaCl, pH 7.4, 5% glycerol, 0.5 mM TCEP, 0.02% DDM) with one pill of complete EDTA-free protease inhibitor cocktail (Roche), and lysed by sonication in an ice-water bath (1 s sonication + 1 s hold, 90% power, for 1 min total; this was repeated 2 times). The whole cell lysates were mixed with 6 ml of 25% Triton X-100 (final concentration of ~4% v/v) and left on an end-over-end rotator at 4 °C overnight.

The lysates were subjected to centrifugation (31,000 × *g* for 45 min) in a JA17 rotor (Beckman Coulter) at 4 °C, and supernatants, containing the proteins of interest, were collected. The raw protein solution was then mixed with 3 ml of pre-washed Ni Sepharose™ 6 Fast Flow resin (Cytiva), and put on an end-over-end rotator at 4 °C for 1 hour. The slurry was transferred to a gravity column (Bio-rad Econo-Pac Chromatography Columns) and allowed to drain. The resin was then washed four times using 15 ml of *washing buffer* (*lysis buffer* with 1% OG and 20/30/40/50 mM incremental imidazole), before elution with 6 ml of *elution buffer* (25 mM HEPES, 500 mM NaCl, pH 7.4, 5% glycerol, 0.5 mM TCEP, 1% OG, 250 or 400 mM imidazole). Protein concentration was determined by staining SDS-PAGE gels with Coomassie Brilliant Blue R-250, using BSA as a standard.

DNA-MSP conjugation

DNA-MSP conjugates were prepared using a previously described protocol, with some modifications[3]. In brief, free amines were removed from amine-oligo (DNA2-5amine) using an Amicon Centrifugal Filter (3k). Then the hetero-bifunctional linker SMCC (5 mM) in DMSO was reacted with amine-oligo (250 μM) in 100 mM phosphate buffer (pH 7.4) for 1 hour at 37 °C. The product (maleimide-oligo) was purified by ethanol precipitation and dissolved in water. Meanwhile, ~80 μM MSP was first treated with 10 times excess of TCEP for 1 hour at 4 °C, then subjected to Amicon Ultra-4 Centrifugal Filter (3k) to remove the TCEP. The DNA-MSP conjugation reaction occurred by mixing maleimide-oligo and TCEP-treated MSP in a 10:1 molar ratio on an end-over-end rotator at 4 °C overnight. Next, the mixture was added to 1 ml of Ni Sepharose™ 6 Fast Flow resin and put on an end-over-end rotator at 4 °C for 1 hour. The slurry was transferred to a gravity column and washed four times using *washing buffer 2* (25 mM HEPES, 140 mM NaCl, 0.5 mM TCEP, pH 7.4). DNA-protein conjugates and unconjugated protein bound to the resin, while unconjugated oligos were washed away. *Elution buffer 2* (*washing buffer 2* with 400 mM imidazole) was added to elute the conjugation products.

Nanodisc preparation

DNA-tethered v-SNARE-reconstituted nanodiscs were produced based on a previous study[3]. DNA-MSP conjugates, v-SNARE protein, and buffer-resuspended lipids were mixed in a final concentration of 4 μM, 15 μM, and 300 μM respectively in 500 μl *hydration buffer* (25 mM

HEPES, 100 mM KCl, 1 mM TCEP, 5% glycerol, pH 7.4) containing 0.06% DDM. After 30 minutes of gentle shaking, 150 µl water-suspended Bio-Bead adsorbents (Bio-Rad) were added to remove DDM during an end-over-end sample mixing overnight at 4 °C. The solution was carefully collected by pipette and purified by a size-exclusion column (Superdex 200 increase 10/300) on a ÄKTA pure chromatography system (Cytiva), using *hydration buffer*.

Liposome preparation

Liposomes were produced by dialysis-based detergent removal[4,7]. First, component lipids were mixed in needed ratio with 1500 nmol lipids in total, nitrogen-dried for 10 minutes, and vacuum-dried for at least 2 hours in a glass tube. The dried lipids were then resuspended in 500 µl *hydration buffer* containing 1.5% OG by vortexing for 10 minutes, followed by adding another 1 ml *hydration buffer* dropwise to bring OG concentration down to 0.5%. The solution was then transferred to a dialysis tubing (MWCO 14kDa, Sigma-Aldrich) and dialyzed against 1.5 liter *hydration buffer* in cold room overnight. Finally, the resultant sample was subjected to centrifugation (368,000 × *g* for 4 hours at 4°C, sw55Ti rotor, Beckman) in a three-layer iodixanol (OptiPrep™) gradient (bottom layer: 1.5 ml sample + 1.5 ml 60% iodixanol, middle layer: 1 ml 20% iodixanol in *hydration buffer*, top layer: 300 µl *hydration buffer*), and 400 µl solution at the interface between the top and middle layer was collected by pipette, aliquoted and stored at -80°C.

For making proteoliposomes for the lipid mixing assay, 15 nmol of v-SNARE (synaptobrevin-2) or 7.5 nmol of co-expressed t-SNARE (syntaxin-1A and SNAP-25B) was included in the original 500 µl *hydration buffer* before resuspension. The rest of the procedures were the same.

DNA tile design and assembly

6HB DNA tile was designed using caDNAno (cadnano.org)[8], and revised from a previous version[9]. To assemble each version of the tile, corresponding oligos were mixed equimolarly to a final concentration of 3 µM in *folding buffer* (5 mM Tris-HCl, 12.5 mM $MgCl_2$, 1 mM EDTA, pH 8.0), then annealed in a PCR thermocycler (Bio-Rad) by a linear cooling step from 95 °C to 20 °C for over 2 hours (-0.1 °C per 10 second). The products were purified by a size-exclusion column (Superdex 200 increase 10/300) on a ÄKTA pure chromatography system, using *hydration buffer* supplemented with 10 mM $MgCl_2$. Final tile concentration was determined by Nanodrop (Thermo Fisher Scientific) and calculated based on its theoretical MW. Samples were stored at -30 °C.

DNA origami design and preparation

DNA origami structures were designed using caDNAno. The origami brick and 16HB rod were *de novo* design, while the hexagon was revised from a previous report[10].

DNA scaffold strands (8,064 or 7,308 nt) were produced using *E. coli* and M13-derived bacteriophages[11]. In brief, *E. coli* K91endA cells were cultured in YT medium supplemented with 5 mM $MgCl_2$ at 37 °C. When OD600 reached 0.4, the bacteria were transformed with p8064 phage, and continued growing at 37 °C for 4 hours. Bacterial cells were then pelleted by centrifugation and ssDNA phages were recovered from the supernatant by PEG precipitation (4% PEG8000, 0.5 M NaCl). After resuspension in 25 mM Tris (pH 8.0) and purification by

centrifugation, p8064 scaffold was isolated from phage by alkaline/detergent denaturation (2 volume of 0.2 M NaOH and 1% SDS, followed by 1.5 volume of 3 M KOAc) and ethanol precipitation. Final ssDNA product was resuspended in 10 mM Tris (pH 8.0) and its concentration was determined by Nanodrop.

To assemble each origami structure, the corresponding scaffold strands (50 nM) were mixed with a selected pool of staple strands (300 nM each) in *folding buffer*, then annealed in a PCR thermocycler by a cooling step from 80 °C to 24 °C for 36 hours (step 1: from 80 °C to 65 °C, -1 °C per 5 min; step 2: from 64 °C to 24 °C, -1 °C per 50 min; step 3: 15 °C hold)[12]. The products were purified by rate-zonal centrifugation in glycerol gradients (from 45% to 15% in *folding buffer*) as described previously (368,000 × *g* for 1 hours at 4 °C, sw55Ti rotor, Beckman)[13]. After removing glycerol by Amicon centrifugal filter (30k), sample was stored at -30 °C.

Liposome coating and clustering by DNA nanostructures

Preformed liposomes were mixed with DNA-chol (DNA1-5chol) in a DNA-chol-to-lipid molar ratio of 1:100, and incubated at 30 °C for 0.5 hour. Pre-assembled 6HB tiles were then added in a tile-to-DNA-chol molar ratio of 1:10, and incubated at 30 °C for 1 hour. No further purification was performed.

For origami-mediated liposome clustering, pre-assembled DNA origami were added to ssDNA-coated liposomes in an origami-to-DNA-chol molar ratio of 1:60 and incubated at 30 °C for 1 hour.

Liposome functionalization and higher-order organization by DNA nanostructures

Tile-coated liposomes generated above were used subsequently for the following steps. For functionalization with SA, DNA-biotin (DNA2-5biotin) was added in a DNA-biotin-to-tile molar ratio of 2:1, and incubated at 30 °C for 0.5 hour. Then SA (Roche) was added in a SA-to-biotin molar ratio of 2:1, and incubated at 30 °C for 1 hour. The sample was subjected to isopycnic centrifugation (368,000 × *g* for 5 hours at 4 °C, sw55Ti rotor, Beckman) in an iodixanol gradient (bottom layer: 150 μl sample + 150 μl 60% iodixanol, top 6 layers: 60 μl 26%/22%/18%/14%/10%/6% iodixanol in *hydration buffer*) for purification[14]. For functionalization with v-SNARE, premade DNA-tethered v-SNARE-reconstituted NDs (DNDv) were added in a DNDv-to-tile molar ratio of ~2:1, and incubated at 30 °C for 1 hour. No further purification was performed for this sample. For origami-templated higher-order organization, pre-assembled origami rods were added in a rod-to-tile molar ratio of ~1:30, and incubated at 30 °C for 1 hour. No further purification was performed for these samples.

Liposome 1D array by C-tile assembly

Preformed liposomes were mixed with C-chol (C-chol) in a C-chol-to-lipid molar ratio of 1:60, then annealed in a PCR thermocycler by a linear cooling step from 48 °C to 20 °C for 18 hours (-0.1 °C per 4 minutes).

Negative-stain electron microscopy

Formvar/carbon-coated copper grid grids (Ted Pella) were glow discharged at 25 mA for 30

seconds by easiGlow (Pelco). 5 µl sample (diluted to 0.05 mM lipid concentration) was pipetted on the glow-discharged EM grid, stained with 1% pH-adjusted uranyl formate for 1 minute, and washed once by *hydration buffer*. Imaging was performed on a Talos F200C 200kV scanning transmission electron microscope.

Cryo-ET

Grid preparation: 200 mesh copper grids with lacey carbon film (Electron Microscopy Science) were glow discharged at 15 mA for 60 seconds in a Pelco easiGlow System (Ted Pella). The grids were loaded into a Vitrobot Mark IV plunge freezer (Thermo Fisher Scientific), with the sample chamber pre-equilibrated to 100% relative humidity at 4 °C. 3 µl sample was pipetted onto a glow-discharged grid, blotted for 2 seconds with filter paper from both sides, and plunge-frozen into liquid ethane.

Tomography data collection and processing: Frozen samples were imaged on Janelia Krios1, a 300 kV Titan Krios cryo electron microscope (Thermo Fisher Scientific) equipped with a high-brightness Field Emission Gun (x-FEG), a spherical aberration corrector, a Bioquantum energy filter and a K3 direct electron detector (Gatan Inc). Tilt series were collected using SerialEM dose-symmetric scheme with a tilt range of +/- 54°, a tilt step of 3°, and grouping of two images on either side (0°, 3°, 6°, -3°, -6°, 9°, 12°, -9°, -12°, …)[15]. Target defocus was set to -2.5 micrometers. The total dose applied to each tilt series was 120 e^-/Å^2. Images acquired on the K3 detector were recorded in super-resolution and dose-fractionation mode (movie frames). The calibrated pixel size was 2.06 Å per physical pixel (1.03 Å per super-resolution pixel) at a nominal magnification of 33kx.

The movie frames were motion-corrected using MotionCor2 without dose weighting[16]. The resulting corrected tilt series were aligned and reconstructed with AreTomo. Tomogram display and 3D modelling was performed using IMOD[17].

Lipid mixing assay

Lipid mixing assays were based on FRET between Rhod-PE and NBD-PE[7]. In brief, 30 µM Rhod/NBD-labelled v-SNARE liposomes (coated or uncoated) and 300 µM t-SNARE liposomes (coated or uncoated) were mixed (1:10 molar ratio) in 100 µl hydration buffer supplemented with 10 mM $MgCl_2$ (no $MgCl_2$ for Figure 5b & S19). Immediately after mixing, fluorescence (excitation: 475 nm, emission: 530 nm) was recorded every two minutes on a plate reader (BioTek Cytation 1, Agilent) at room temperature. After 1.5 hours, 2 µl intervention sample (ssDNA or DNase I) was added, and fluorescence was monitored for another 1.5 hours. Finally, 3 µl 20% OG was added, and fluorescence was measured for another 15 minutes. The final plot was normalized by the smallest (0%) and largest (100%) value of each trace.

Supplementary Figures

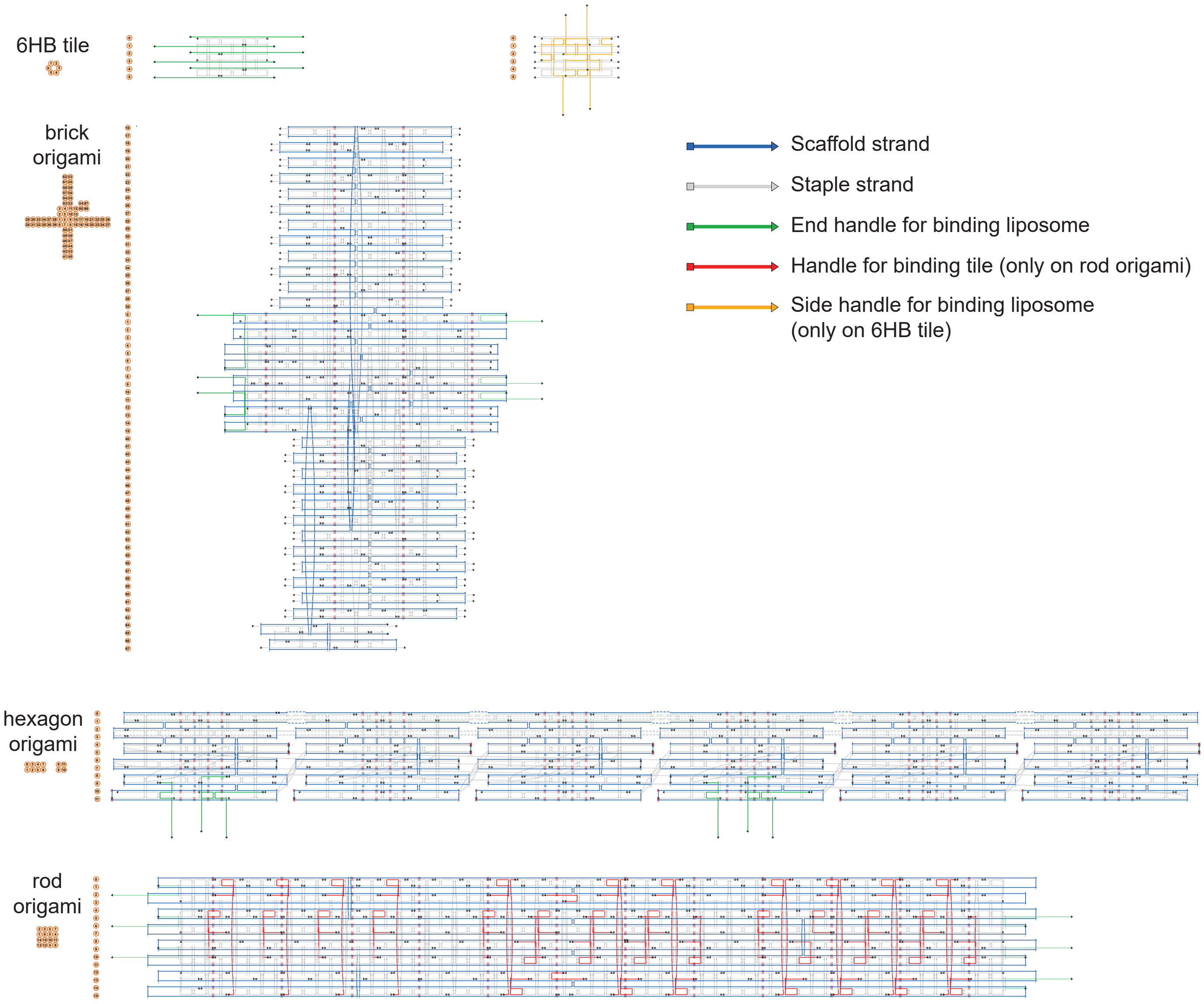

Supplementary Figure 1. CaDNAno design of DNA nanostructures[8]. The 6HB tile is a revised version of a previous design[9]. Two versions of tile are shown: one with 3 handles (green) on each end (left, Fig. 2a), and the other with 2 handles (gold) on each of the two opposite sides (right, Fig. 3a). Other versions of the tile used in this study could be modularly designed by simply changing the number and position of handles. All the three origami structures have 3 handles (green) on each of the two opposite sides, used for clustering liposomes (Fig. 3). The hexagon origami is a revised version of a previous design[10]. The rod origami also has handles (red) for higher-order organization of tile-coated liposomes (Fig. 4). A selective subgroup of these handles was used in each version, to control the placement of liposomes. The scaffold strand of the 180-nm rod is p8064, while the scaffold strand of the 60-nm brick and 80-nm hexagon is p7308. CaDNAno files and sequences of DNA nanostructures are provided online.

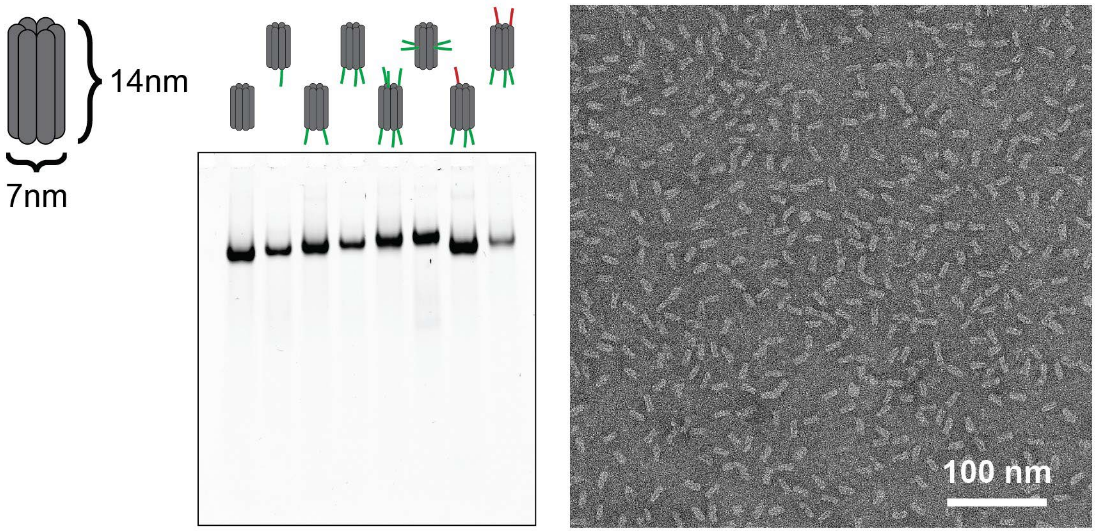

Supplementary Figure 2. Gel (6% native PAGE in 1×TAE with 10 mM $MgCl_2$) and negative-stain (n.s.) TEM characterization of 6HB DNA tiles. Structure shapes shown in the EM image were consistent with the design. A single major band for each version of the 6HB tile appeared in native PAGE, validating its purity and homogeneity. A GeneRuler™ Low Range DNA Ladder (Thermo Scientific, #SM1191) is used as reference in the gel.

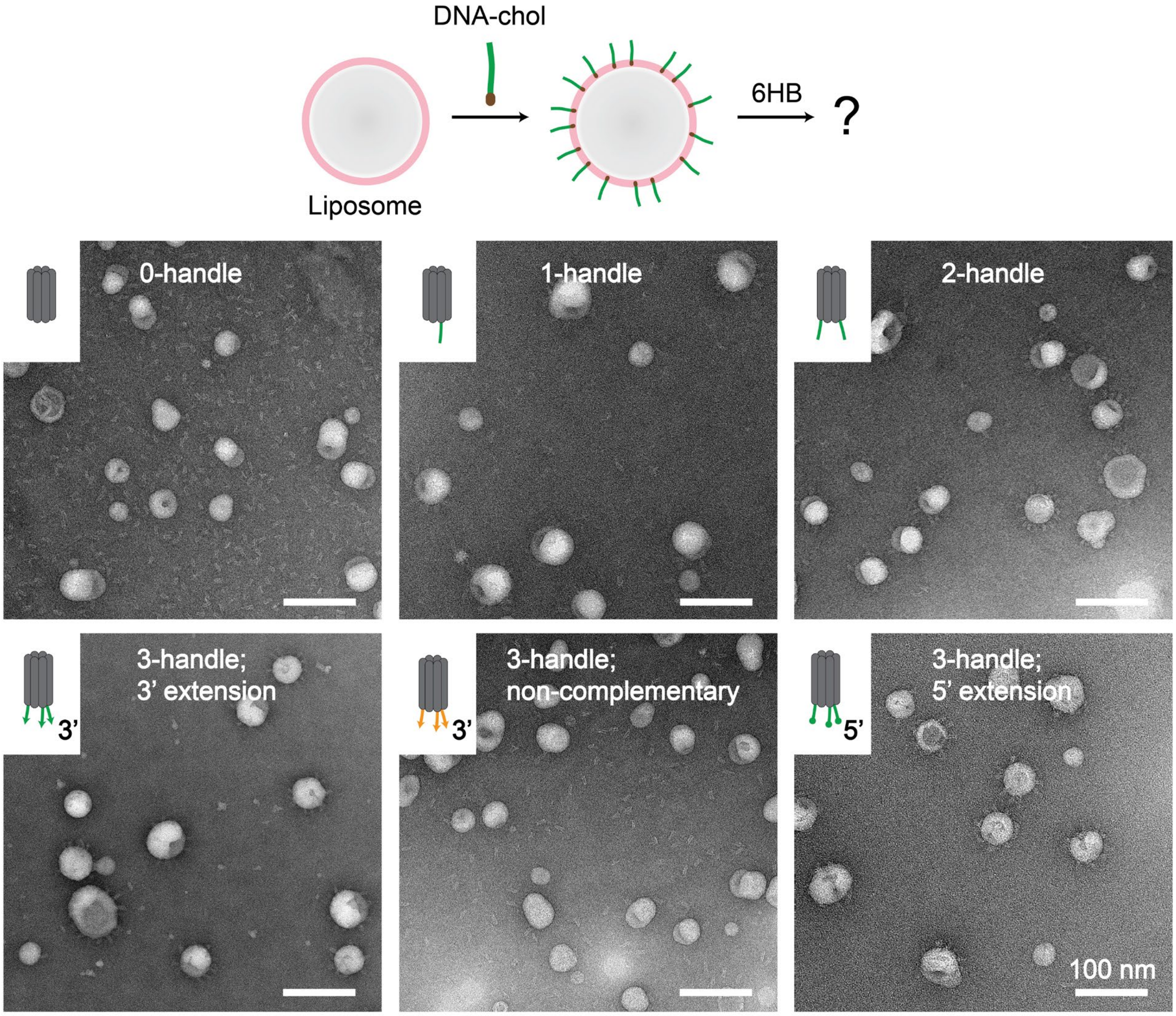

Supplementary Figure 3. The copy number and sequence of ssDNA handles on each 6HB tile affecting liposome coating efficiency, evaluated by negative-stain TEM images. Preformed liposomes were treated with DNA-chol in a DNA-chol-to-lipid molar ratio of 1:100, then the corresponding 6HB tile was added. The results show that tiles with two handles (top right) coated liposomes as efficiently as tiles with three handles (bottom left), while tiles with one handle were less efficient since some unattached tiles could be seen (top middle). No attachment was observed for tiles without handles (top left) or with three non-complementary handles (bottom middle), ruling out the presence of nonspecific binding due to electrostatic interactions[18]. In all cases, handles were extended from the 3' ends of oligos. When three handles were extended from the 5' end (bottom right), tiles also bound to liposomes effectively. Scale bars: 100 nm.

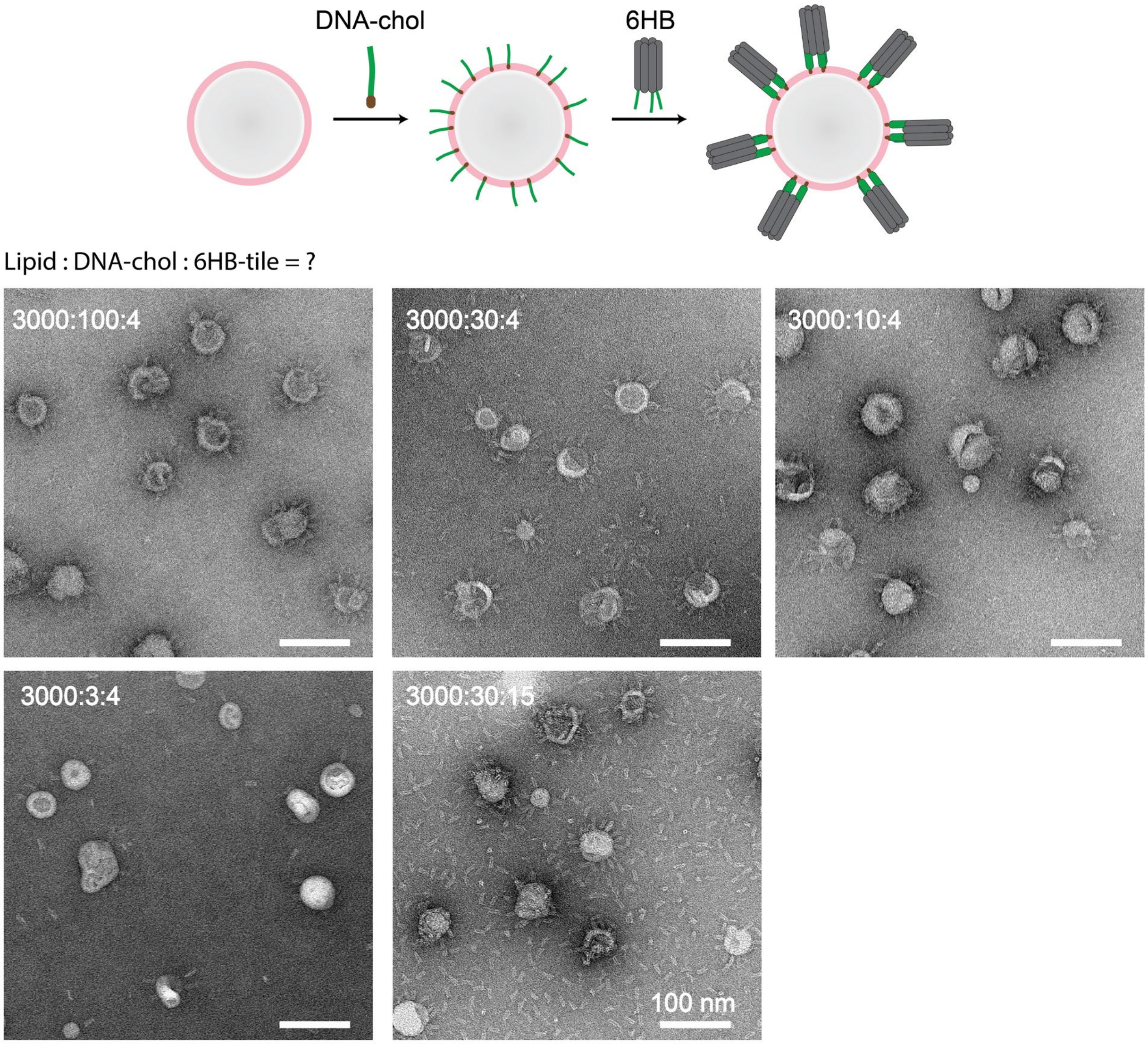

Supplementary Figure 4. The molar ratio of lipid to DNA-chol to 6HB-tile (marked on each representative negative-stain TEM image) affecting liposome coating efficiency. Preformed liposomes were first treated with DNA-chol, then 6HB tiles with 3 handles on one end. When the molar ratio of lipid to tile was kept at 3000:4, a 100:4 (top left), 30:4 (top middle), and 10:4 (top right) molar ratio of DNA-chol to tile were all capable of mediating tile-liposome association, while a 3:4 ratio (bottom left) resulted in low binding efficiency. This suggests that the concentration of DNA-chol was critical to stabilize tile binding on liposomes. Compared to 3000:30:4 (top middle) which was used as a standard ratio in this study, a 3000:30:15 molar ratio (bottom middle) lead to many unattached tiles lying in the background, probably because the liposome surfaces have been saturated by tiles already. Scale bars: 100 nm.

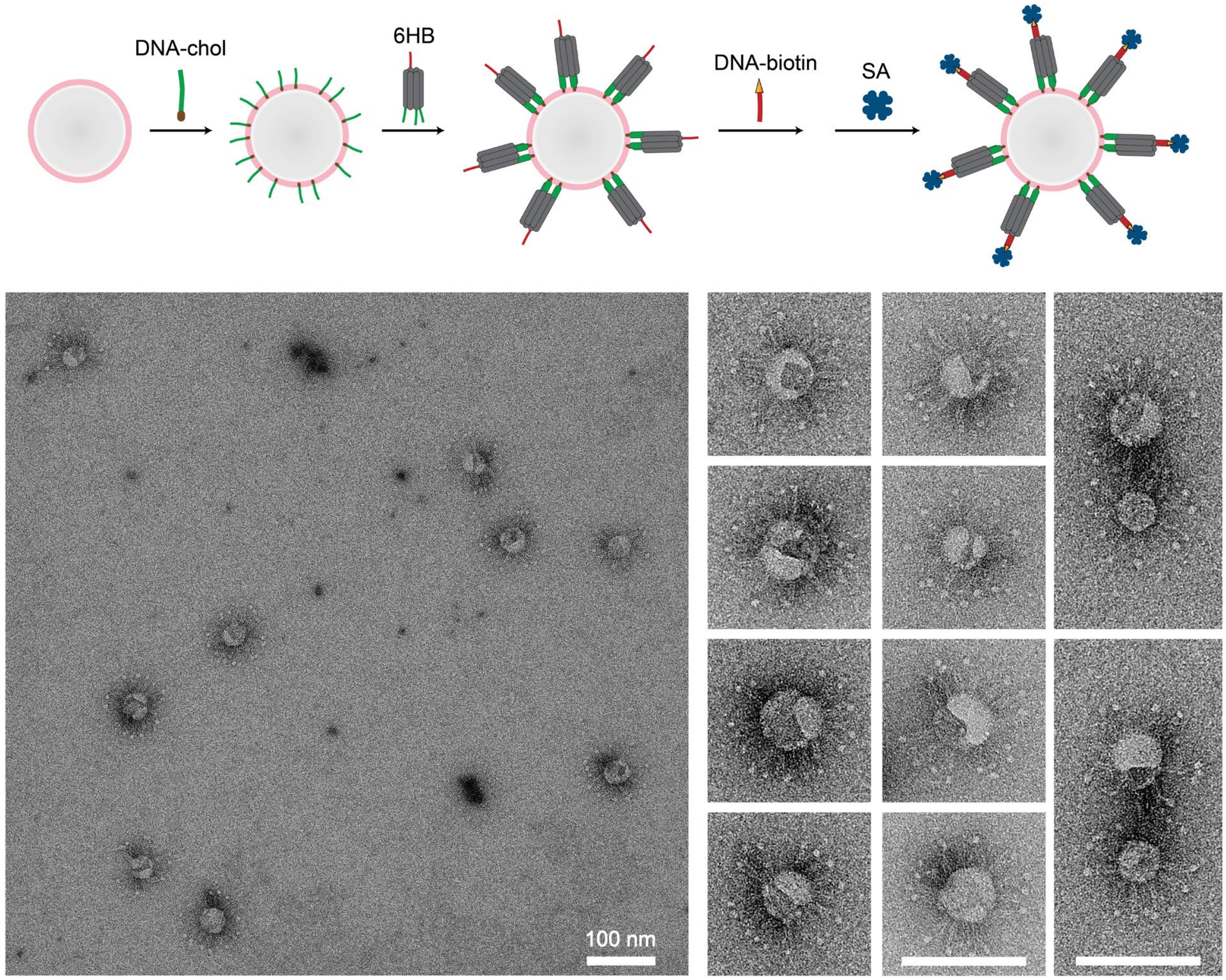

Supplementary Figure 5. Cartoon model and negative-stain TEM images of tile-mediated display of streptavidin (SA) on liposomes. Preformed liposomes were mixed with DNA-chol (DNA1-5chol), 6HB tile, DNA-biotin (DNA2-5biotin), and SA sequentially, followed by an isopycnic centrifugation purification. Core-satellite liposome-SA complexes were observed, and tiles serving as intermediary adaptors were distinguishable in the EM images. Although most of the complexes were separated, some of them coupled into oligomers (right column), probably due to the tetravalency of SA. Scale bars: 100 nm.

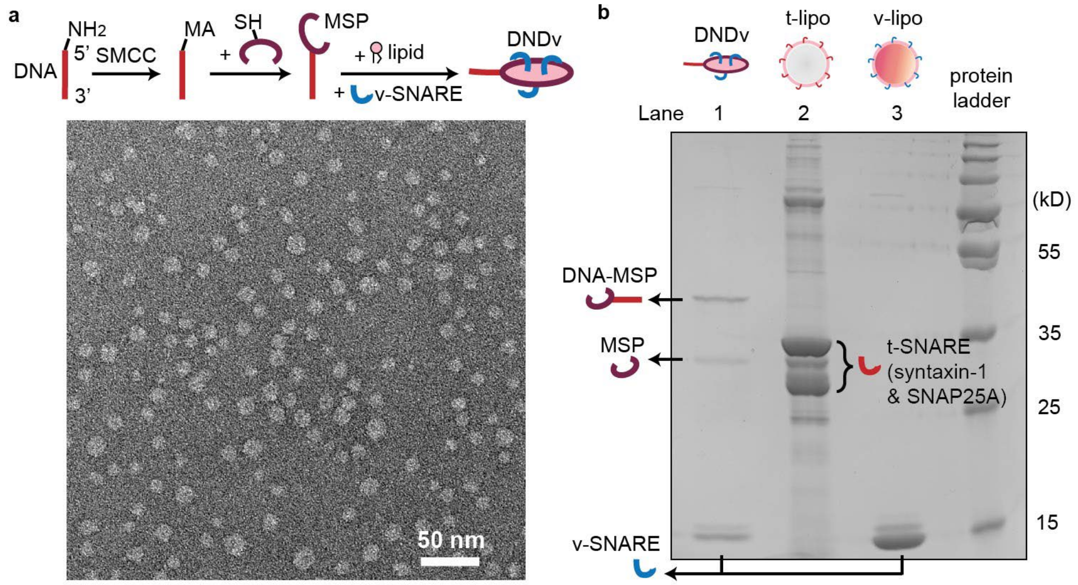

Supplementary Figure 6. a. Schematic and TEM image of DNA-tethered v-SNARE-reconstituted ND (DNDv). The yield of DNA-MSP conjugation was ~70% (calculated by conjugated MSP divided by total MSP) (data not shown), and the size and shape of DNDv under EM were as expected (~14 nm in diameter). **b.** Denaturing PAGE gel (4% stacking, 12% resolving) of DNDv and SNARE-reconstituted liposomes. The band at the bottom of lane 1 confirmed the incorporation of v-SNARE in DND (six v-SNAREs in each ND on average). The two main bands in lane 2 and one main band in lane 3 showed the presence of t-SNARE and v-SNARE in corresponding liposomes.

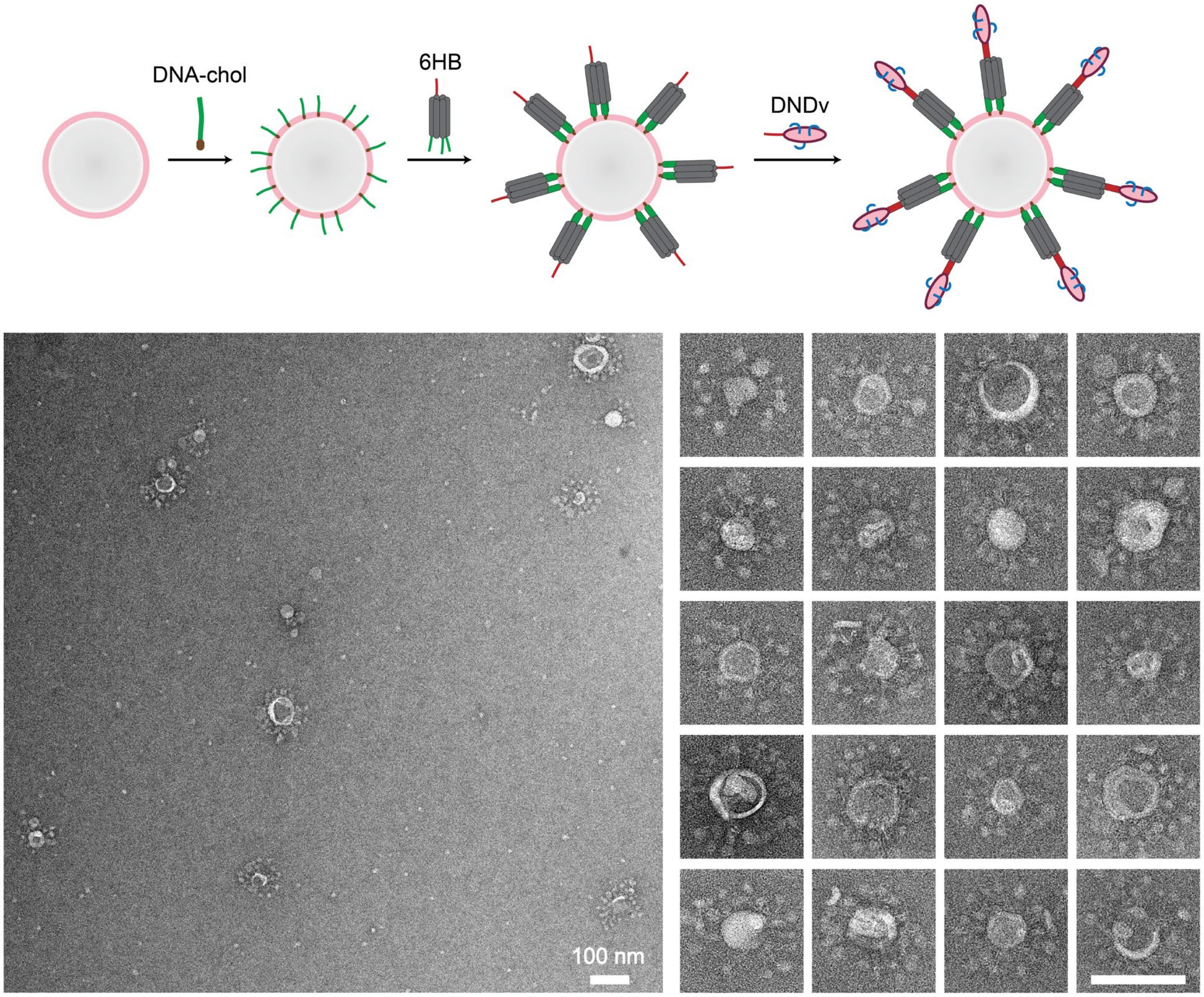

Supplementary Figure 7. Cartoon model and negative-stain TEM images of tile-mediated display of v-SNARE on liposomes. Preformed liposomes were mixed with DNA-chol (DNA1-5chol), 6HB tile, and DNDv (DNA2-5amine) sequentially before EM imaging. Core-satellite liposome-DNDv complexes were observed, and tiles serving as intermediary adaptors were distinguishable in EM images. It is worth noting that this is the first nanostructure constructed from two widely used model membrane systems: liposomes and NDs. Scale bars: 100 nm.

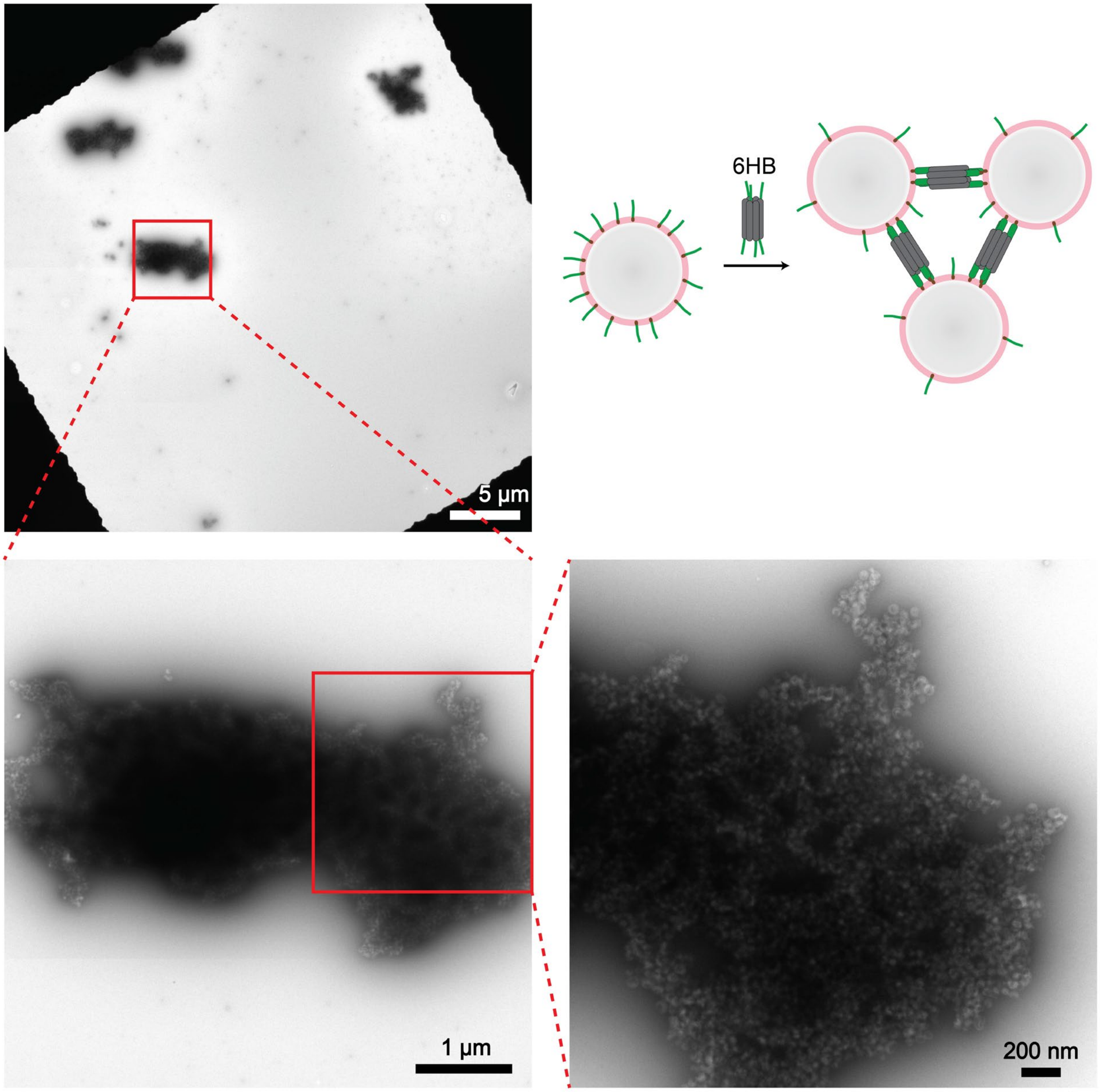

Supplementary Figure 8. Serial zoom-in negative-stain TEM images of tile-mediated liposome clusters. Typically, liposome clusters (0.05 mM lipid concentration) absorbed to random positions on EM grids and could be easily spotted in zoom-out views. Cluster size ranged from sub-micrometer to over 10 micrometers, and the shape also varied significantly. In close-up views, the center of the cluster was often too dark to see clearly, probably due to stacked liposomes. As a result, many of the negative-stain TEM images were taken at the edge of each cluster.

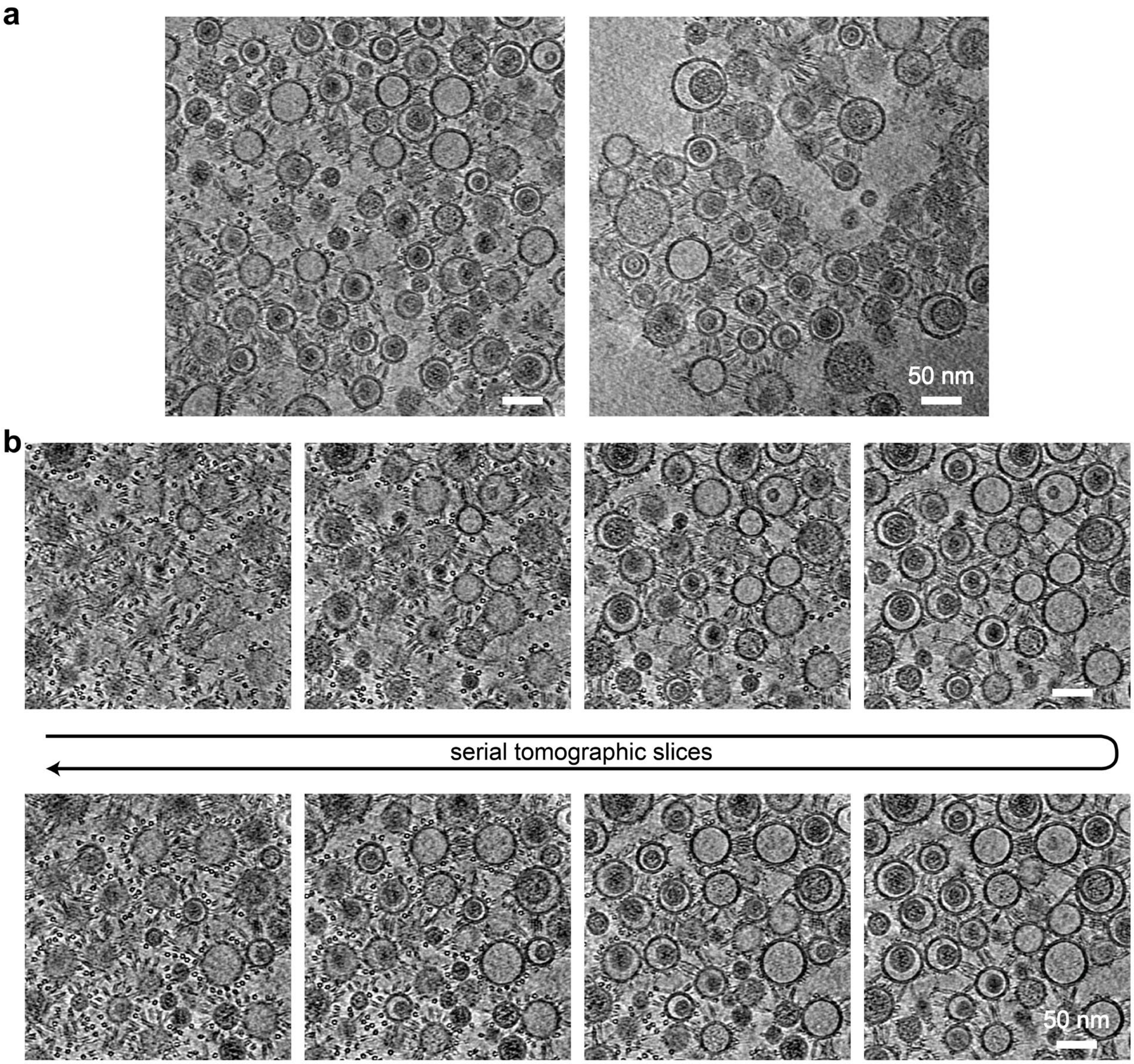

Supplementary Figure 9. Cryogenic electron tomographic (cryo-ET) slices of tile-mediated liposome clusters. **(a)** Two typical liposome clusters. **(b)** Serial tomographic slices of another liposome cluster. Compared to potential deformation during sample preparation for negative-stain EM, samples were frozen in amorphous ice for cryo-ET, which in principle preserved the 'native' shape and configurations. As expected, liposome networks formed by tiles were observed, and the length of the tiles between neighboring liposomes were consistent with the designed value (~30 nm). Lipid bilayers of individual liposomes remained intact and spherical shapes, indicating that the attaching and linking by DNA nanostructures did not impair the membrane. We note that some of the liposomes contained another vesicle inside, which was interesting but not relevant to the goal of this study. A tomogram video is available online. We also note that higher electron density can be seen in some of the vesicles, which might be caused by the iodixanol used to purify liposomes via isopycnic centrifugation. Scale bars: 50 nm.

a

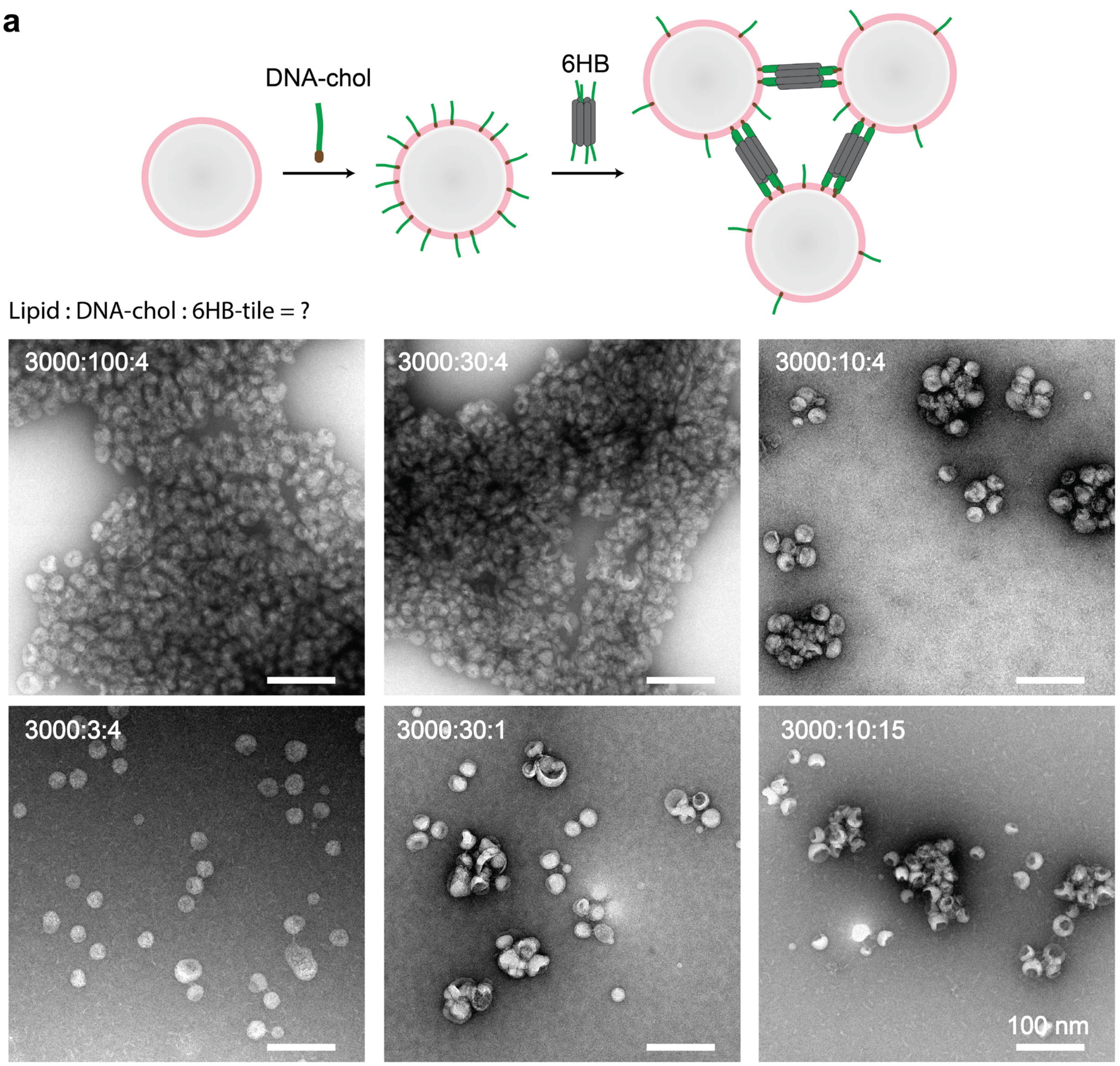

b

liposome diameter	n.s. TEM	p (t-test)	cryo TEM
uncoated liposome	72 ± 14	N/A	
coated liposome	66 ± 14	0.01	
clustered liposome	58 ± 13	2E-6	53 ± 8
reversed clustered liposome	69 ± 14	0.29	

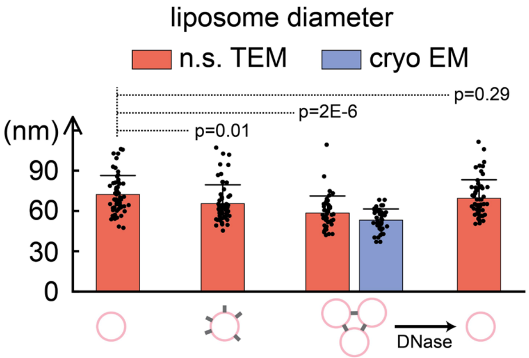

Supplementary Figure 10. a. The molar ratio of lipid to DNA-chol to 6HB-tile (marked on each representative negative-stain TEM image) affecting liposome clustering efficiency. Preformed liposomes were first treated with DNA-chol, then 6HB tile with 3 handles on both ends. When the

molar ratio of lipid to tile was kept at 3000:4, both 100:4 (top left) and 30:4 (top middle) molar ratio of DNA-chol to tile were efficient in mediating liposome clustering, while a 3:4 ratio (bottom left) can only form liposome oligomers (note the unbound tiles lying in the background). A 10:4 molar ratio of DNA-chol to tile (top right) generated small liposome clusters, and adding more tiles (10:15) did not make the cluster bigger (bottom right). On the other hand, when the molar ratio of lipid to DNA-chol was kept at 3000:30, a 30:1 molar ratio of DNA-chol to tile (bottom middle) formed much smaller clusters than the standard 30:4 ratio (top middle). Taken together, the concentration of both DNA-chol and tiles were critical to, and could be used to tune, the size of the liposome clusters formed in this system. **b.** Liposome diameters measured in negative-stain TEM images, showing that after clustering is reversed by DNase treatment, liposome sizes remain unchanged compared to the sizes before clustering (69 ± 14 vs. 72 ± 14 nm; $p = 0.29$ by two-tailed t-test). We note that liposomes are partially flattened on negative-stain TEM grids. For example, the measured diameter of liposomes within clusters is bigger in negative-stain TEM images than cryo-EM images (58 ± 13 vs. 53 ± 8 nm). Interestingly, DNA tiles, coated on the liposome surface, can help maintain the spherical shape of vesicles (i.e., they become less flattened) on TEM grids (66 ± 14 vs. 72 ± 14 nm; $p = 0.01$ by two-tailed t-test). N = 58, 59, 43, 58 for negative-stain EM in each case from left to right. N=45 for cryo EM. Source data are provided as a Source Data file. Mean diameter values with standard deviations are listed in the table (left) and are plotted in the bar graph with error bars (right). Scale bars: 100 nm.

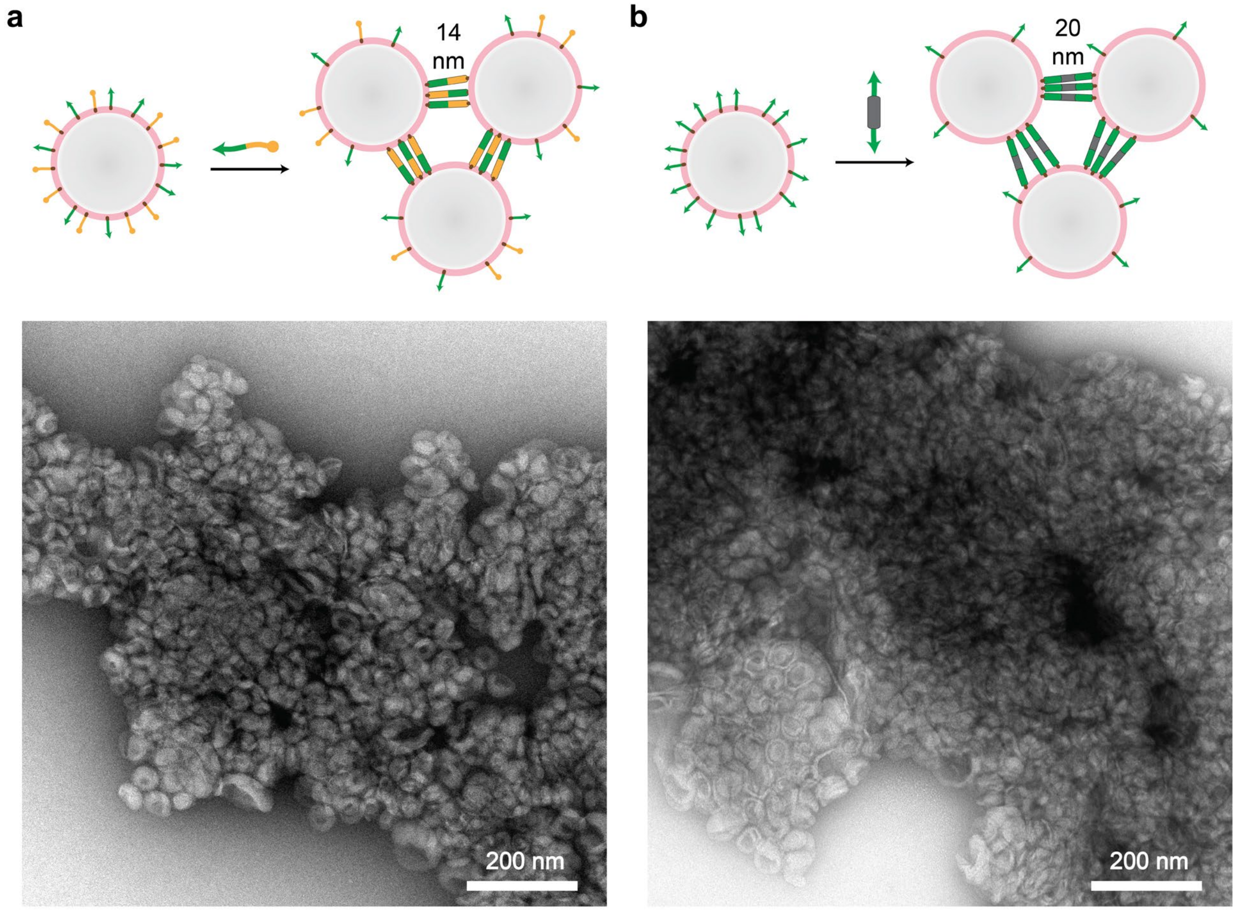

Supplementary Figure 11. Cartoon model and negative-stain TEM images of liposome clustering mediated by ssDNA **(a)** or dsDNA **(b)**. **a.** Because the two complementary strands of a DNA double helix are antiparallel, two different versions of DNA-chol with the cholesterol modified at the 3' (arrow) or the 5' (dot) end respectively are required for a ssDNA linker to function. Here, preformed liposomes were mixed with 5'-modified DNA-chol (DNA1-5chol) and 3'-modified DNA-chol (DNA2-3chol), after which a ssDNA linker (linker_12) complementary to both DNA-chol strands was added. With a lipid-to-DNA-chol-to-linker molar ratio of 3000:50:50, large liposome clusters were formed. **b.** Preformed liposomes were mixed with 5'-modified DNA-chol (DNA1-5chol), after which a preassembled two-strand dsDNA linker with one ssDNA extension at each end (linker_ds1 + linker_ds1') was added. With a lipid-to-DNA-chol-to-linker molar ratio of 3000:30:15, large liposome clusters were formed.

In both clusters formed above, liposomes were tightly packed without obvious sign of fusion. The theoretical length of the final DNA duplex (after hybridization with DNA-chol) was 14 nm **(a)** and 20 nm **(b)** respectively, but the dsDNA between liposomes was too small to visualize in these TEM images. Scale bars: 200 nm.

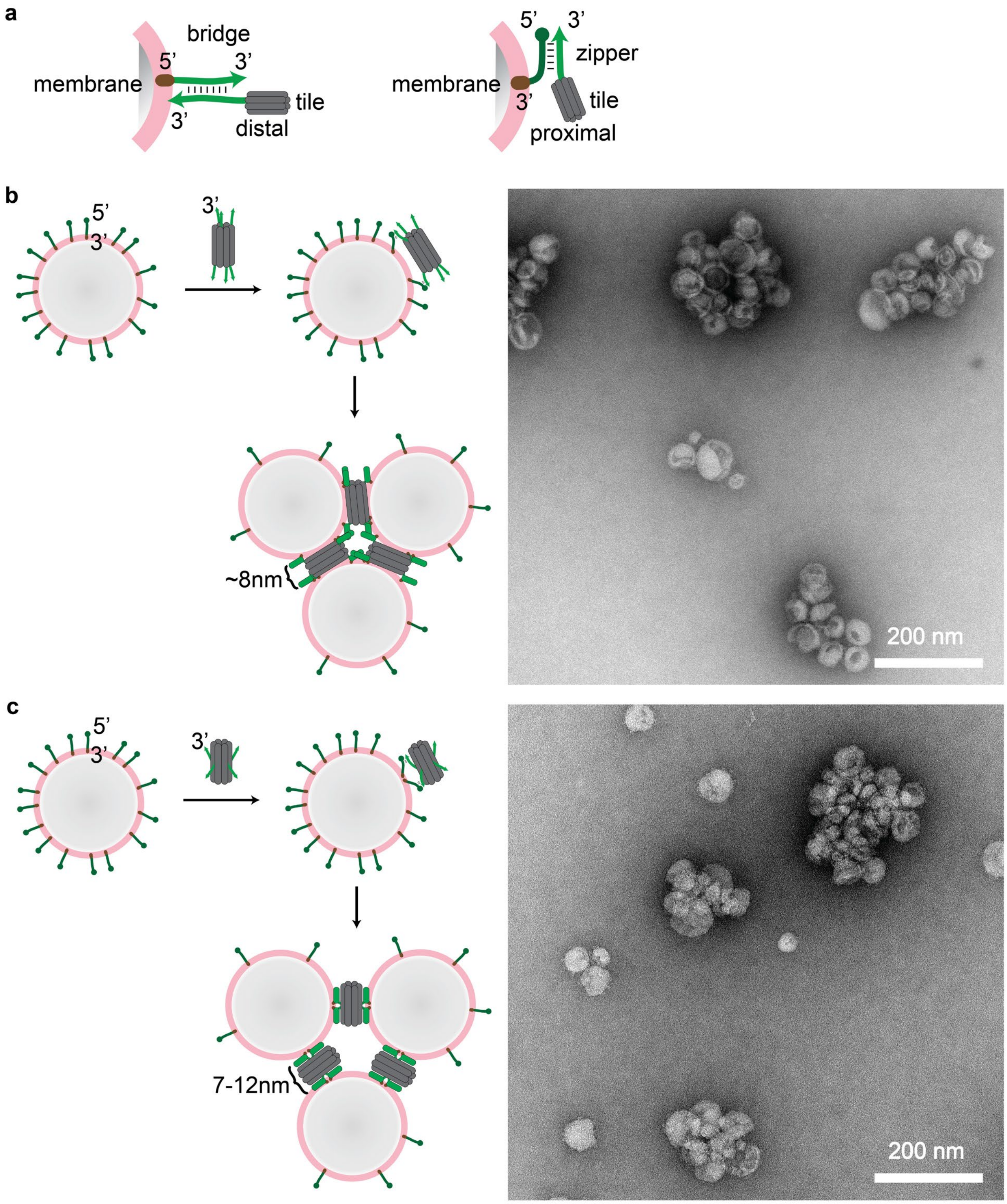

Supplementary Figure 12. Cartoon model and negative-stain TEM images of liposome clustering mediated by 6HB tiles and 3'-modified DNA-chol. The two complementary strands of a DNA double helix run in antiparallel directions. As a result, a 5'-modified DNA-chol hybridizes with a 3'-extended handle in a 'bridge' conformation, causing the tile to be 'distal' to the membrane surface (**a,** left), while a 3'-modified DNA-chol hybridizes with a 3'-extended handle in

a ‘zipper’ conformation, potentially pulling the tile more proximal to the membrane (**a,** right). For most of this study, we used 5’-modified DNA-chol, thus adopting a bridge conformation. Here we tested the feasibility of using 3’-modified DNA-chol with a zipper conformation. The advantage of this configuration is that shorter membrane intervals could be achieved, e.g., ~8 nm when tiles with end handles are used (**b**).

Here, preformed liposomes were mixed with 3’-modified DNA-chol (DNA1-3chol), then either end-handle tiles (**b**) or side-handle tiles (**c**) were added. Negative-stain TEM showed liposome clusters were formed in both cases. As expected, liposomes were tightly associated in these clusters, although precise measurement of interval distance was difficult due to vesicle flattening and overlapping on the substrate. The cluster sizes here were generally smaller than the ones with bridge conformations. This reduced linking efficiency might arise from the repulsion between negatively-charged DNA tiles and DOPS-contained membrane. Scale bars: 200 nm.

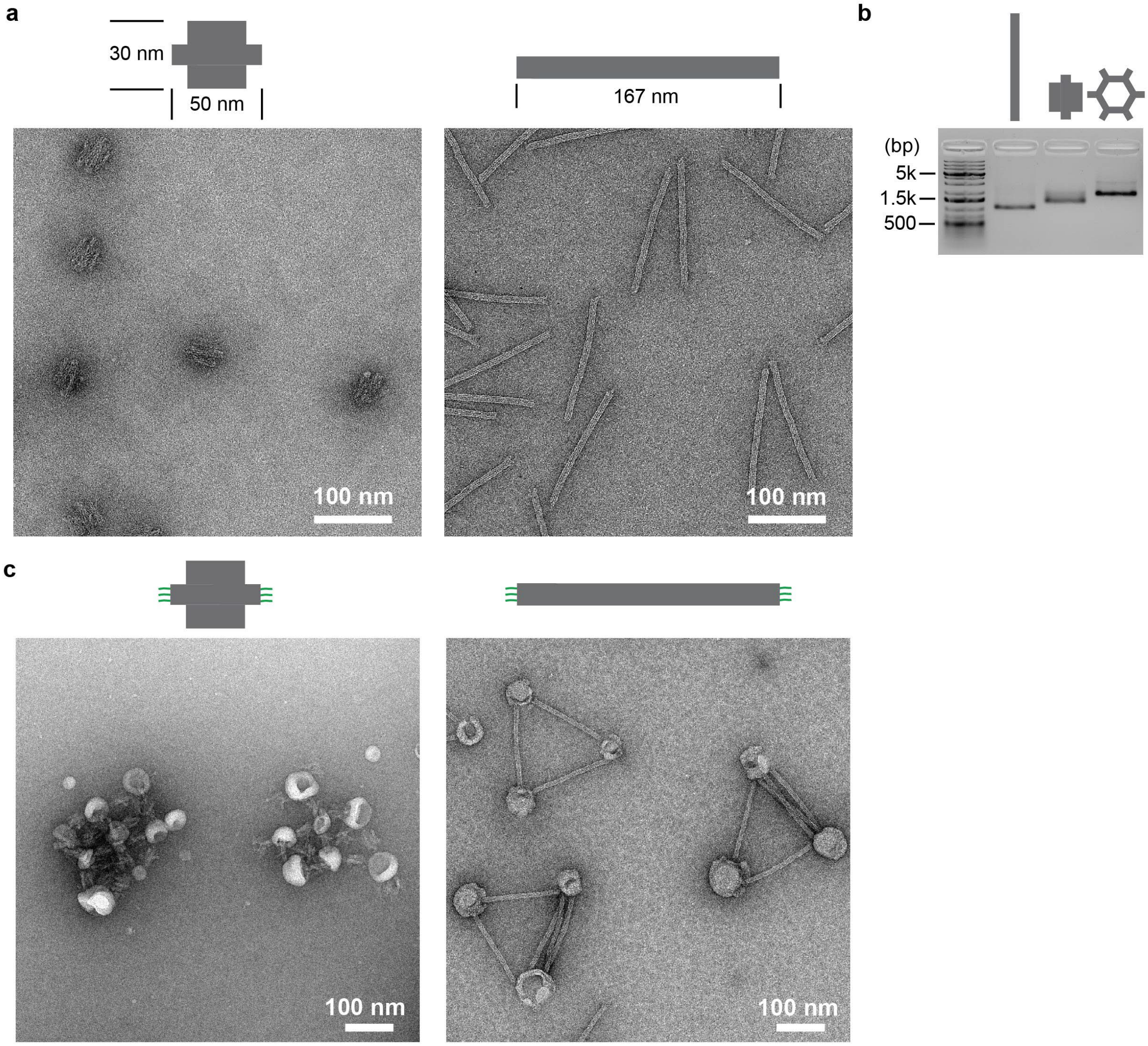

Supplementary Figure 13. Negative-stain TEM images (**a**) and gel characterization (**b**, 1.5% agarose with 10 mM $MgCl_2$) for DNA origami. Theoretical lengths were marked on each cartoon model. Structures generally folded well and had good purity after rate-zonal centrifugation. A GeneRuler™ 1kb Plus DNA Ladder (Thermo Scientific, #SM1331) is used as reference in the gel. **c.** Origami-mediated liposome patterns. Preformed liposomes were mixed with DNA-chol, then origami bricks (left) or rods (right) with 3 handles on each of the two ends were added in an origami-to-DNA-chol-to-lipid molar ratio of 1:120:12000. Compared to the clusters formed in the standard 2:120:12000 ratio (Fig. 3), the liposome networks generated here were smaller. Interestingly, triangular liposome patterns were enriched in the products, likely because the triangle is the strongest shape. Scale bars: 100 nm.

a

(nm)	n.s. TEM	cryo TEM
30-nm tile	16 ± 2	29 ± 2
20-nm tile	8 ± 2	
60-nm rod origami	47 ± 9	
180-nm rod origami	168 ± 5	

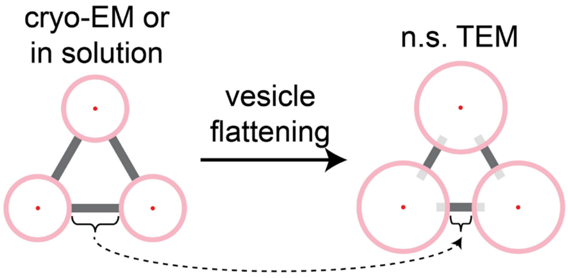

b

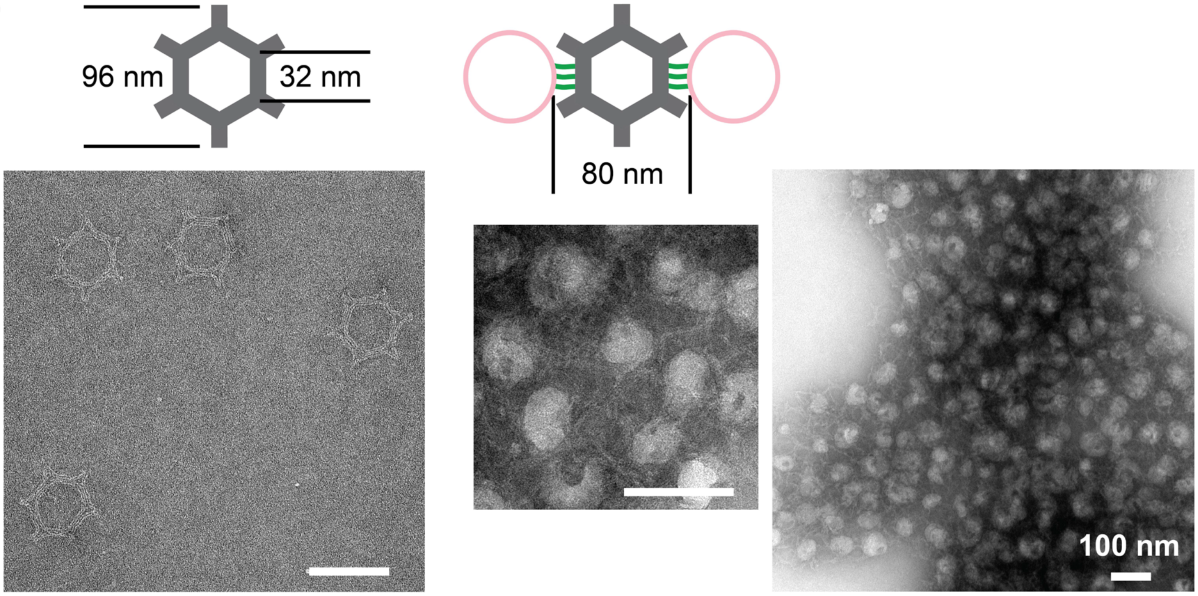

Supplementary Figure 14. a. Inter-membrane distances within DNA-mediated liposome clusters measured in EM images. Only distinguishable liposomes near the edge of the cluster were analyzed. More than 20 distances were measured for each sample. Average values and standard deviations are listed in the table. With the 30-nm 6HB tile as linker, the distances measured via cryo EM (29 ± 2 nm) match the design well, while the distances measured via n.s. TEM (16 ± 2 nm) are significantly shorter. Two major reasons account for this discrepancy: (i) Individual liposomes are flattened in n.s. TEM images (also see Figure S10b), thus shortening the gap between two neighboring liposomes (see cartoon model on the right); (ii) a cluster in the n.s. TEM image is actually the 2D projection of a partially collapsed 3D solution structure. Similarly, the measured inter-membrane distances in other liposome clusters (mediated by 20-nm tile, 60-nm rod origami, or 180-nm rod origami) in n.s. TEM are also 12-15 nm shorter than the theoretical value. **b.** Cartoon model and negative-stain TEM images of a hexagon DNA origami used for liposome clustering. The measured inter-membrane distance is 48 ± 10 nm, which is far less than the design (80 nm), indicating low rigidity of this framework structure. Scale bars: 100 nm.

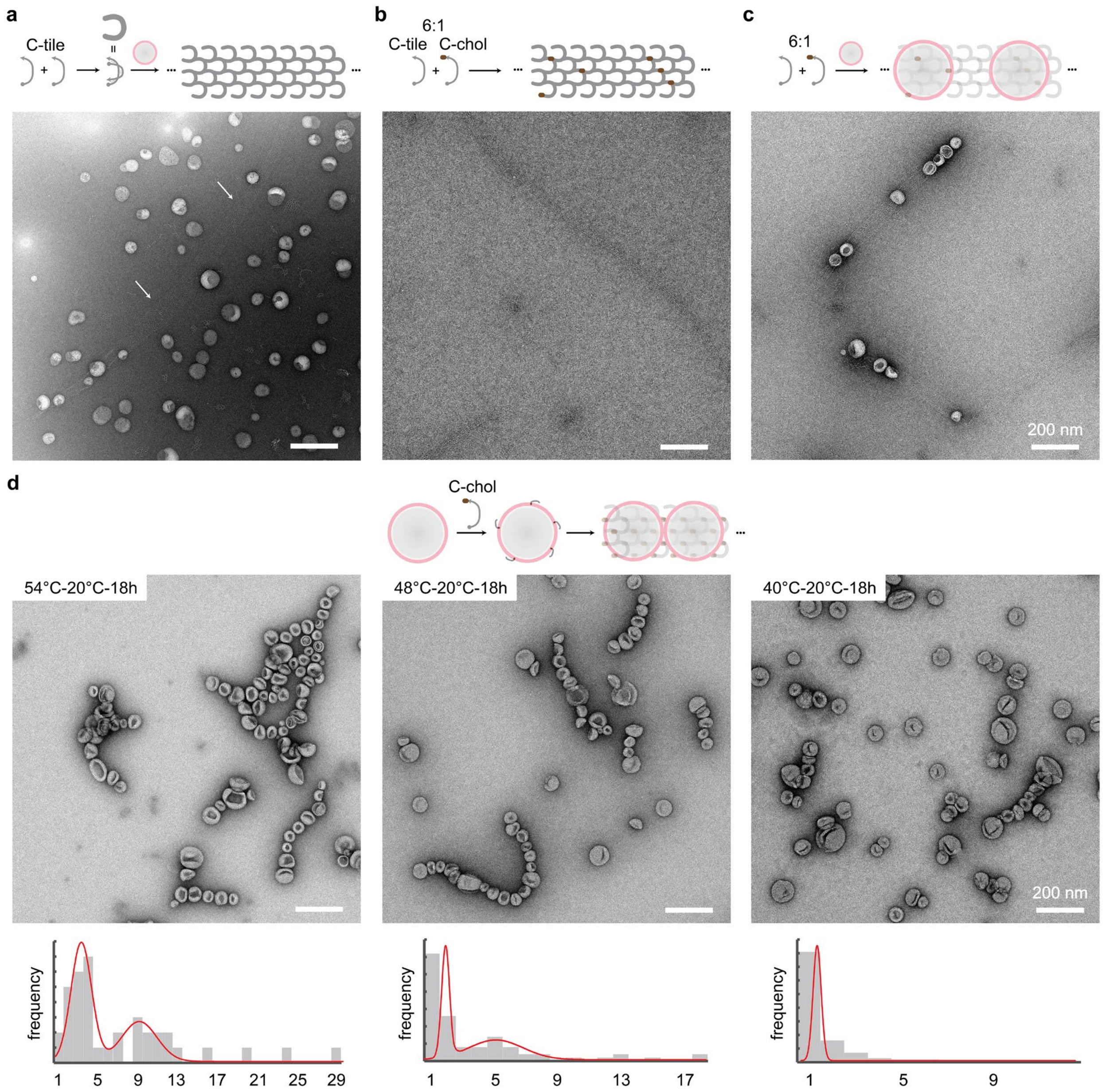

Supplementary Figure 15. Schematics and negative-stain TEM images of C-tile assembly and templated liposome array. The sequence of the C-tile (C-tile) has self-complementary domains, which allows two C-tiles to fold into one C-shaped motif (**a**), which in turn can form tubes or 2D arrays[1]. In the original study, the assembly was performed by a one-pot annealing from 95 °C to 22 °C for over 48 hours in a water bath[1]. To prevent potential damage to liposomes from heat, we decreased the initial temperature to 48 °C, and shortened the total annealing time to 18 hours. **a.** Under a 48°C-20°C-18h annealing process, C-tiles tended to form micrometers-long, sub-100-nm-wide ribbons (white arrows), which were slightly different from the tube-like structures observed in the previous report[1]. Liposomes were included in this reaction, and as expected, they did not associate with the ribbon due to the lack of membrane anchors. **b.** When cholesterol-modified C-tiles (C-chol) were mixed with unmodified C-tiles in a 1:6 molar ratio,

narrower ribbons were formed compared to the products of only using unmodified C-tiles. **c.** When liposomes were included in the reaction of (**c**), they bound to the ribbon, forming discontinuous 1D arrays. The underlying DNA ribbons were visible, corroborating the DNA template effect on liposome string formation. **d.** Effect of starting temperature in an 18-h annealing protocol (ending temperature kept at 20 °C) when only C-chol is used. A representative TEM image and a histogram of liposome counts in a string are shown for each starting temperature (54 or 48 or 40 °C). In all cases, 1D liposome strings along with isolated liposome individuals and clusters coexist in the products. Apparently, higher starting temperature (e.g., 54 °C) leads to strings composed of more liposomes (9 vs. 5 liposomes per string), but includes more aggregates. 48°C-20°C-18h is an optimal annealing protocol for enriching liposome strings. Scale bars: 200 nm.

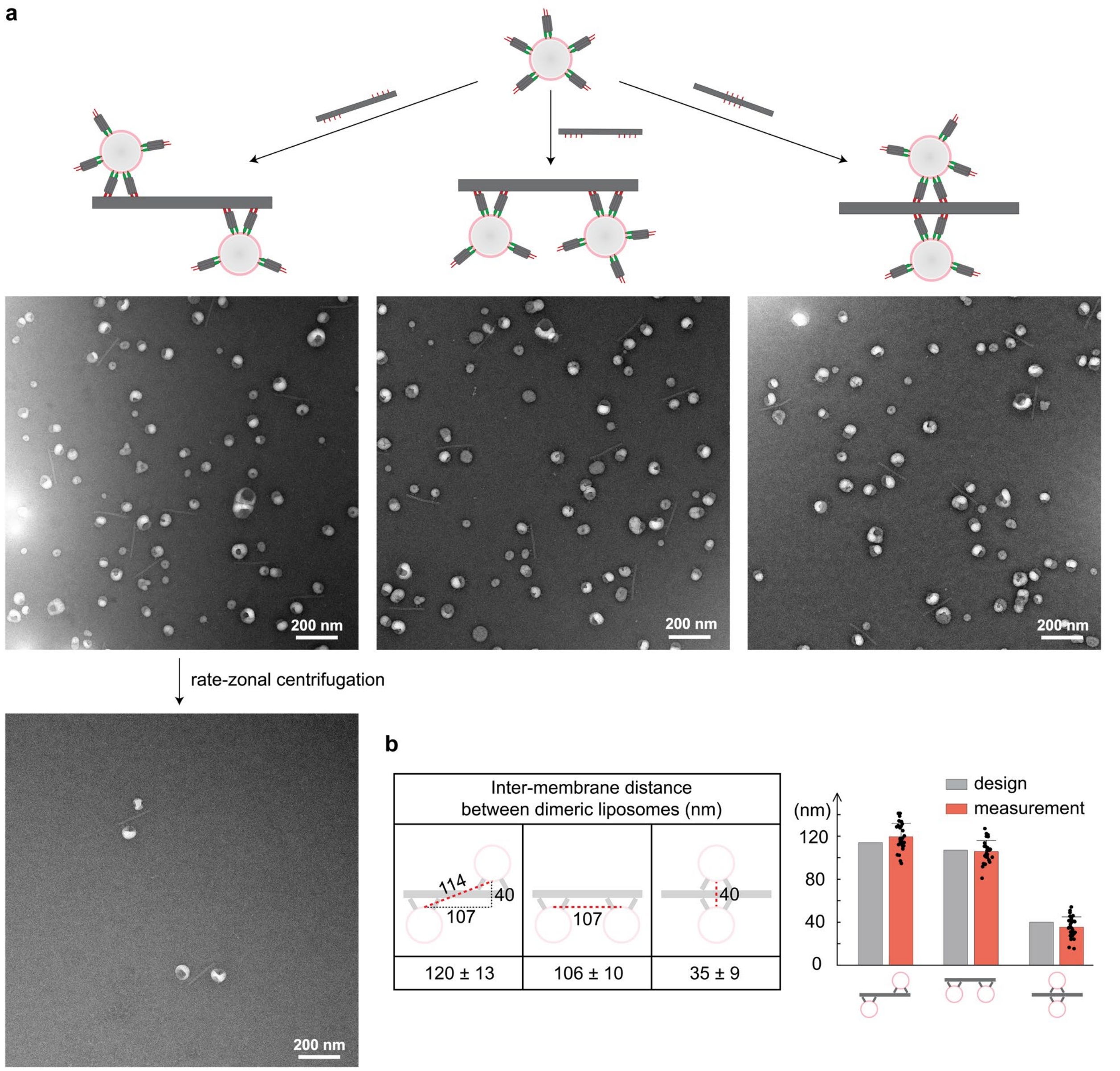

Supplementary Figure 16. a. Cartoon model and negative-stain TEM image of tile-coated liposome duos organized by origami rods. Preformed liposomes were mixed with DNA-chol, 6HB tile with different handles at the two ends, and one version of origami rod sequentially. Each rod has two groups of 4 handles (red), which can hybridize with their complementary handles on the tiles, enabling organization of two tile-coated liposomes. Each handle group on a rod can only host one liposome, due to size restriction. Three different versions of rods were assembled and used for templating liposome dimers with distinct distances and configurations. TEM images confirmed most of the rods had two liposomes attached at the desired positions where tiles were also visible, agreeing with our design. Excessive liposomes are required to saturate the binding sites on origami; optionally, they can be removed by rate-zonal centrifugation (15%-45% glycerol, 368,000 × *g*, 1 h, 4 °C)[13], as demonstrated using one of the configurations (bottom left). Scale bars: 200 nm. **b.** Theoretical and measured inter-membrane distance between

origami-templated dimeric liposomes. N = 30, 31, 32 for each configuration from left to right. Source data are provided as a Source Data file. Average values and standard deviations are shown in both the table (left) and bar graph with error bars (right). In general, the liposome distances are consistent with the designs.

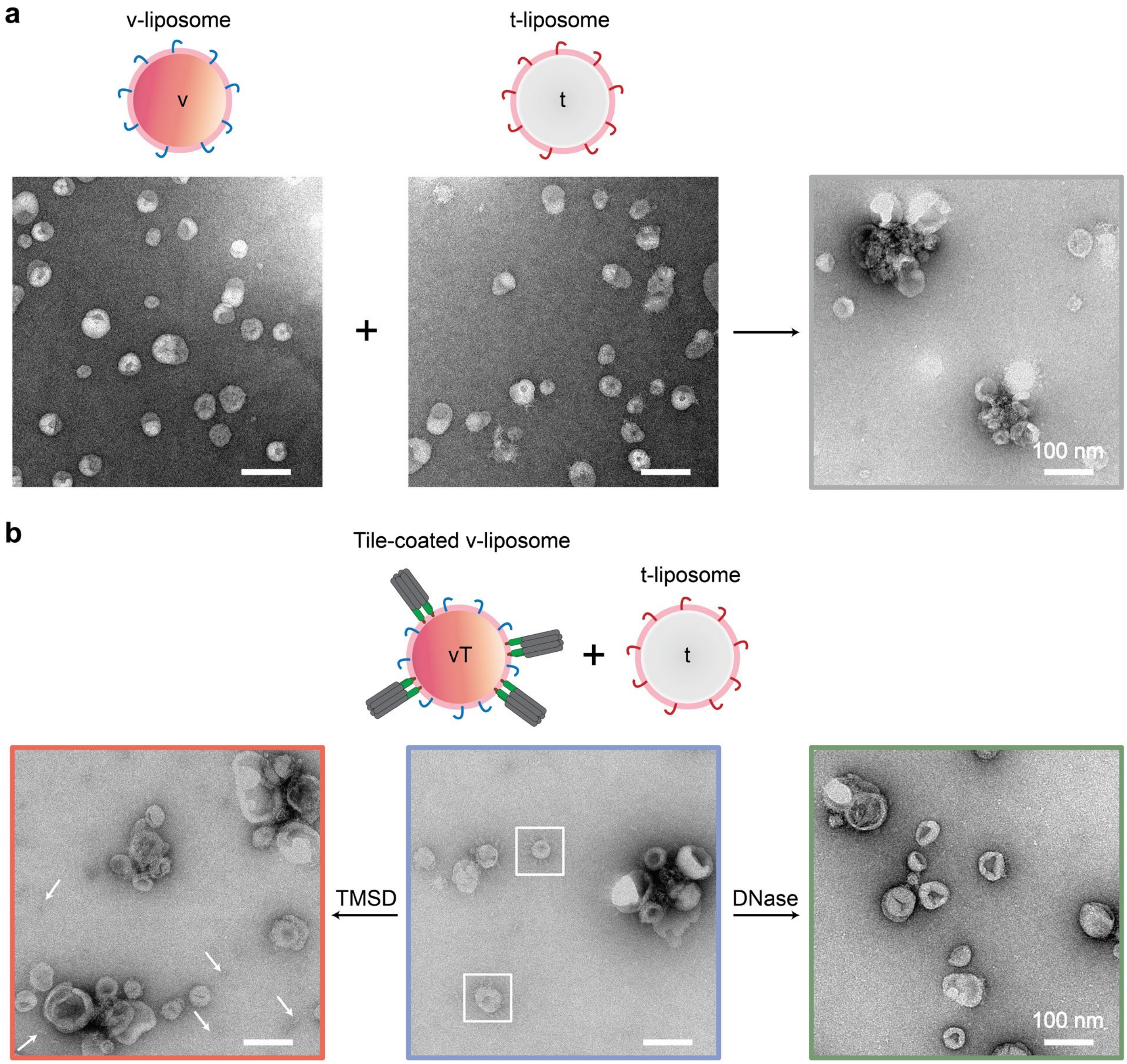

Supplementary Figure 17. Negative-stain TEM images showing uncoated **(a)** or tile-coated **(b)** v-SNARE liposomes reacting with uncoated t-SNARE liposomes. The outline of the TEM Image of each sample is matched by color in Fig. 5a. **a.** Standard v-SNARE-reconstituted liposomes (left) and t-SNARE-reconstituted liposomes (middle) were generally monodispersed. After mixing them for 3 hours, small liposome clusters were formed (right), likely as the products of SANRE docking and zippering. **b.** When the v-SNARE liposomes were coated with 6HB tiles, the tiles hindered SNARE-mediated t-SNARE liposome docking, as evidenced by isolated tile-studded liposomes in the TEM image (middle, highlighted by white box). After adding DNase (right) or displacing strand for TMSD (left), tiles were removed from the v-SNARE liposomes surface (white arrows pointing at detached tiles after TMSD), presumably allowing SNARE-mediated fusion to proceed.

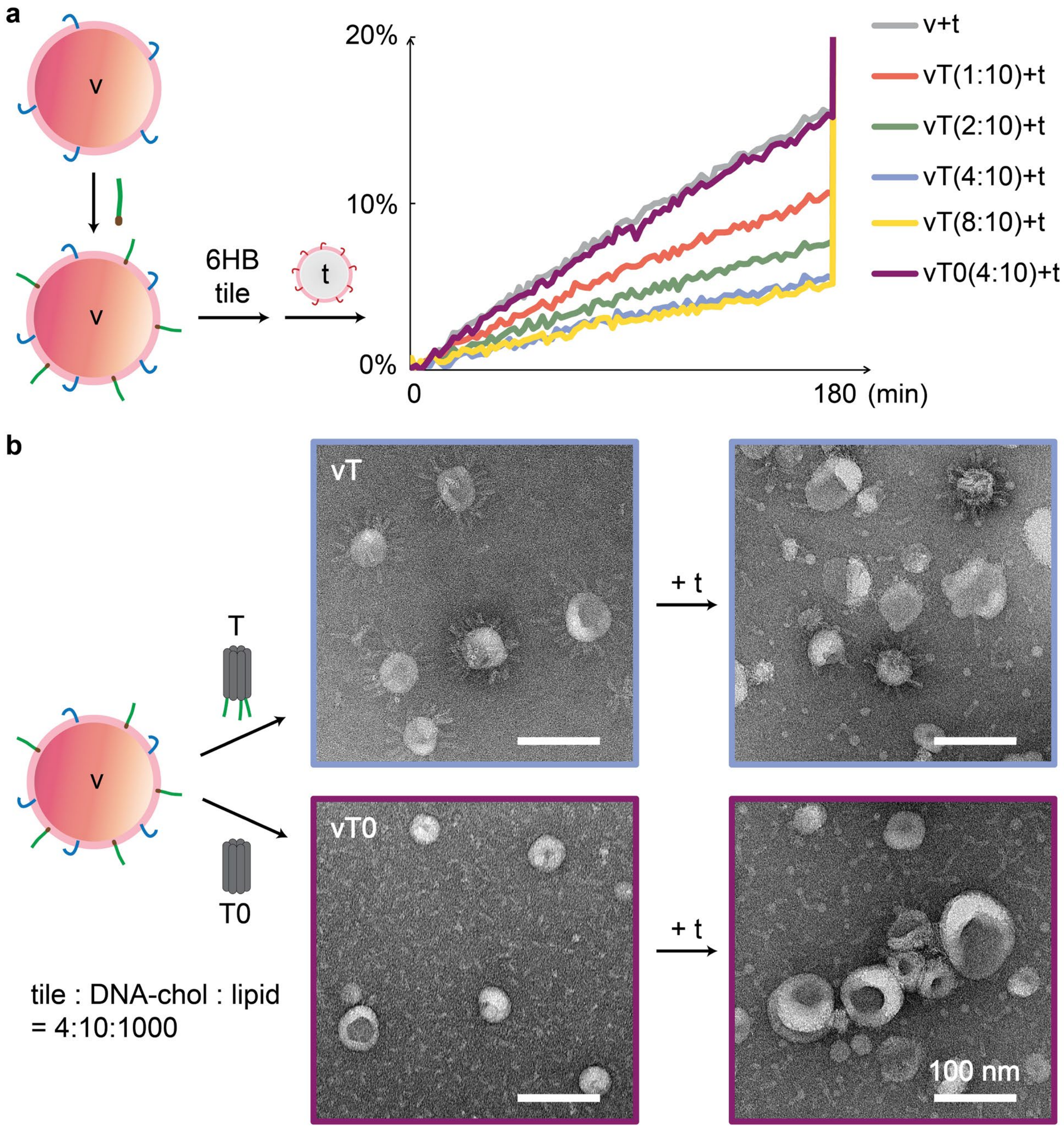

Supplementary Figure 18. Titration of tile coating on v-SNARE liposomes for SNARE-mediated fusion. **a.** V-SNARE liposomes were mixed with DNA-chol, then a series of concentrations of 6HB tiles with 3 handles at one end were added. After incubation at 30 °C for 1 hour, t-SNARE liposomes (v:t=1:10) were added and fluorescence of lipid mixing assay was recorded at room temperature for 3 hours. The ratio shown in the plot legend represents the value of tile:DNA-chol. Normalized curves showed that the more tiles there were, the more significantly SNARE-mediated fusion was suppressed (red, green, blue). The trend stopped at a threshold point (4:10, blue), after which addition of tiles had no further effect on fusion (8:10, yellow). We suspect the liposome surfaces were saturated with tiles at the threshold concentration, and

therefore extra tiles would not affect coating. Adding the threshold concentration of a tile without handles (purple) resulted in the same fusion rate as uncoated v-SNARE liposomes (grey), indicating that tiles in solution did not impact fusion. In other words, membrane surface modification was essential in fusion suppression. **b.** Negative-stain TEM images of two of the samples in **(a)** with the same color code, both before (left) and after (right) adding t-SNARE liposomes. Top panel: v-SNARE liposomes coated with 3-handle tiles remained isolated after adding t-SNARE liposomes. Bottom panel: Tiles without handles did not bind to v-SNARE liposomes (visible in the background) and did not interfere with SNARE-mediated liposome docking. Scale bars: 100 nm.

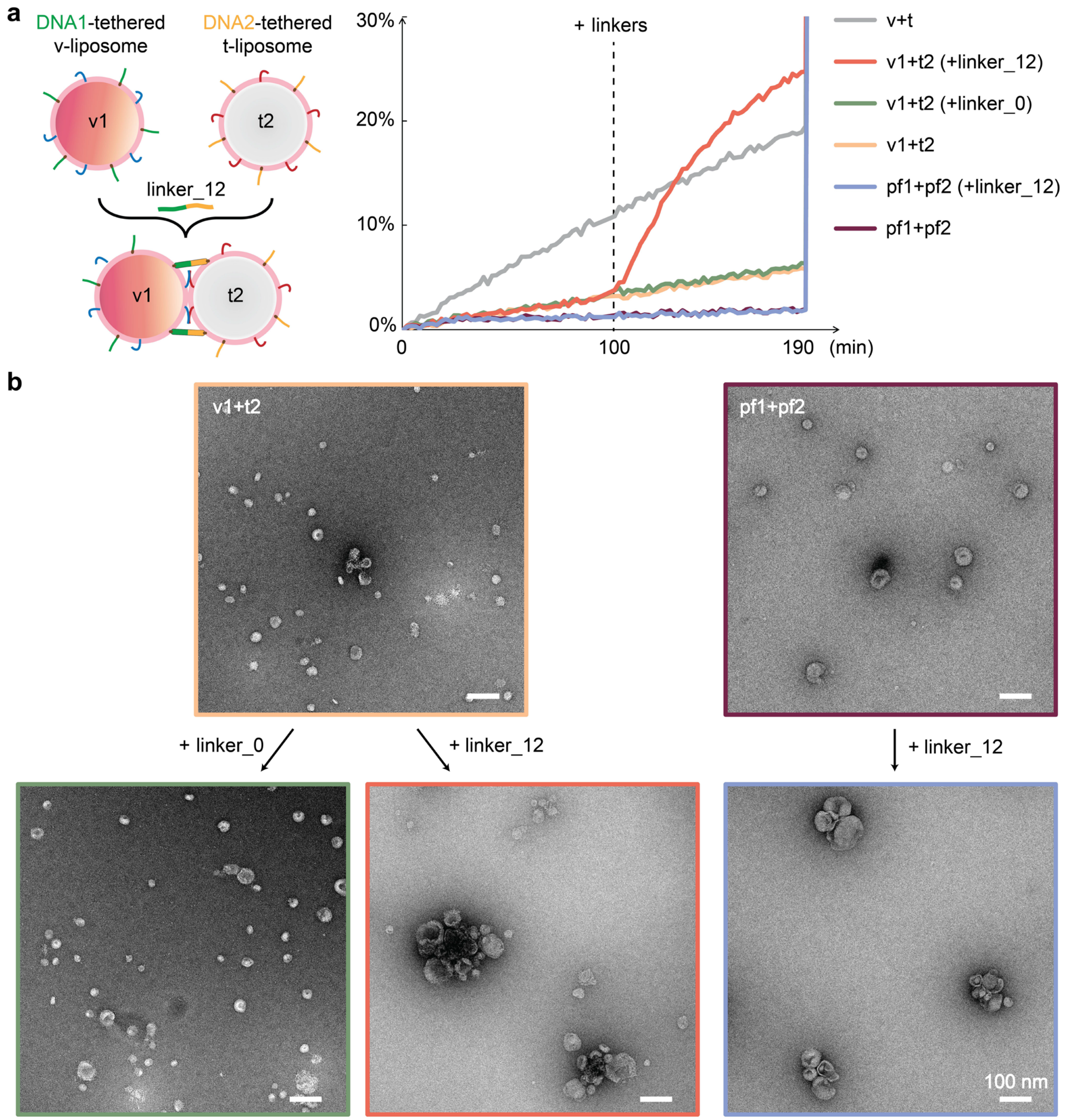

Supplementary Figure 19. Linker-promoted SNARE-mediated liposome fusion. **a.** V- and t-SNARE liposomes were tethered with DNA1-5chol (v1) and DNA2-3chol (t2) respectively in a DNA-chol-to-lipid molar ratio of 200:1, then mixed together in a v1-to-t2 ratio of 1:10 for lipid mixing assay. A ssDNA linker was added to some of the samples at 110 minutes, in a linker-to-DNA1-to-DNA2 ratio of 100:15:150. When the linker was complementary to both DNA1 and DNA2 (linker_12), SNARE-mediated fusion was greatly boosted (red vs. yellow curve), while it did not affect protein-free (pf) liposomes with the same lipid composition and DNA coating (blue vs. purple). However, when the linker was non-complementary to DNA1 and DNA2 (linker_0), fusion between v- and t-SNARE liposomes was unaffected (green vs. yellow). These results

suggested that DNA linking itself would not induce fusion, probably because the membranes were not pulled close enough in the 'bridge' conformation[19], but the linking considerably increased SNARE-driven fusion, probably by tethering v- and t-SNARE liposomes together (similar to Rab proteins as membrane organizers in cells) to help SNARE zippering. **b.** Negative-stain TEM images of some of the samples in **(a)** with the same color code. Liposomes were indeed clustered when linker_12 was added (bottom middle and right), validating the linking effect. Scale bars: 100 nm.

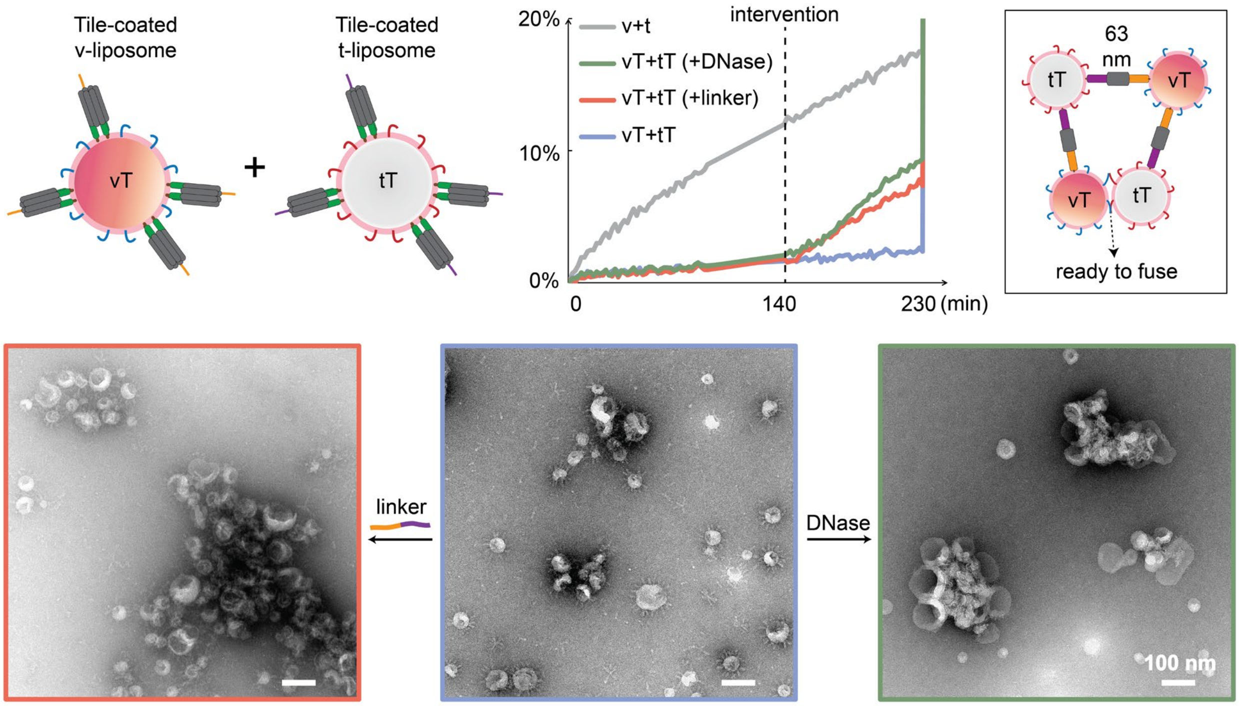

Supplementary Figure 20. Lipid mixing assay and negative-stain TEM images of linker-triggered SNARE-mediated fusion of tile-coated liposomes. The fluorescence curve and EM image outlines are matched by color for the same sample. Both v- and t-SNARE liposomes were coated with 6HB tiles, yet they contained distinct outward handles (gold and purple line at the end of the tile). The acquired tile-coated v-SNARE liposome (vT) and tile-coated t-SNARE liposome (tT) were then mixed together in a 1:10 ratio. EM images confirmed the coating was efficient (bottom middle), which almost completely inhibited SNARE-mediated fusion as shown by the lipid mixing assay results (blue vs. grey curve). Upon addition of DNase, the coatings were degraded, leaving v- and t-SNARE liposomes free to fuse (green curve and bottom right image). Finally, when linkers (complementary to both gold and purple handles on the two types of tiles) were added to the mixture of vT and tT, large liposome clusters emerged in the EM image (bottom left), and fusion activity was rescued (red curve). We note that the total lengths of two linked tiles (~63 nm) is already longer or on par with the liposome diameter (~53 nm measured by cryo-EM). How such a DNA construct facilitates, rather than obstructs, membrane fusion is unclear. One likely explanation is that during the formation of linker-mediated liposome clusters, sometimes SNAREs between a v-/t-SNARE liposome pair can interact before the DNA constructs settle into place (see schematic on the upper right), locally promoting SNARE-mediated docking and zippering to increase the fusion rate. Hence, while the tiles can inhibit fusion between some v- and t-SNARE-bearing liposomes, our observations indicate that the net effect in the entire network is to promote their fusion. This setup, a combination of tile coating and linking, achieved blocking and triggering of SNARE-mediated liposome fusion in a one-pot reaction. Scale bars: 100 nm.

a

(nm)	Dialysis	50-nm extrusion	200-nm extrusion
Liposome diameter	72 ± 14	112 ± 38	199 ± 62

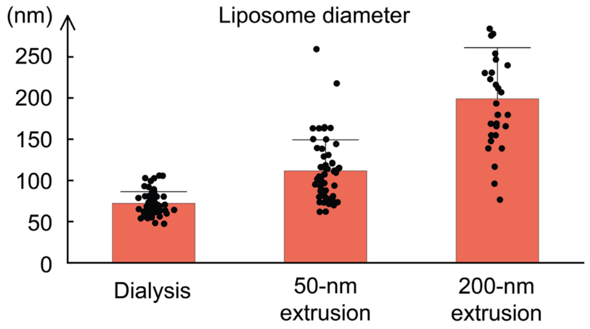

b

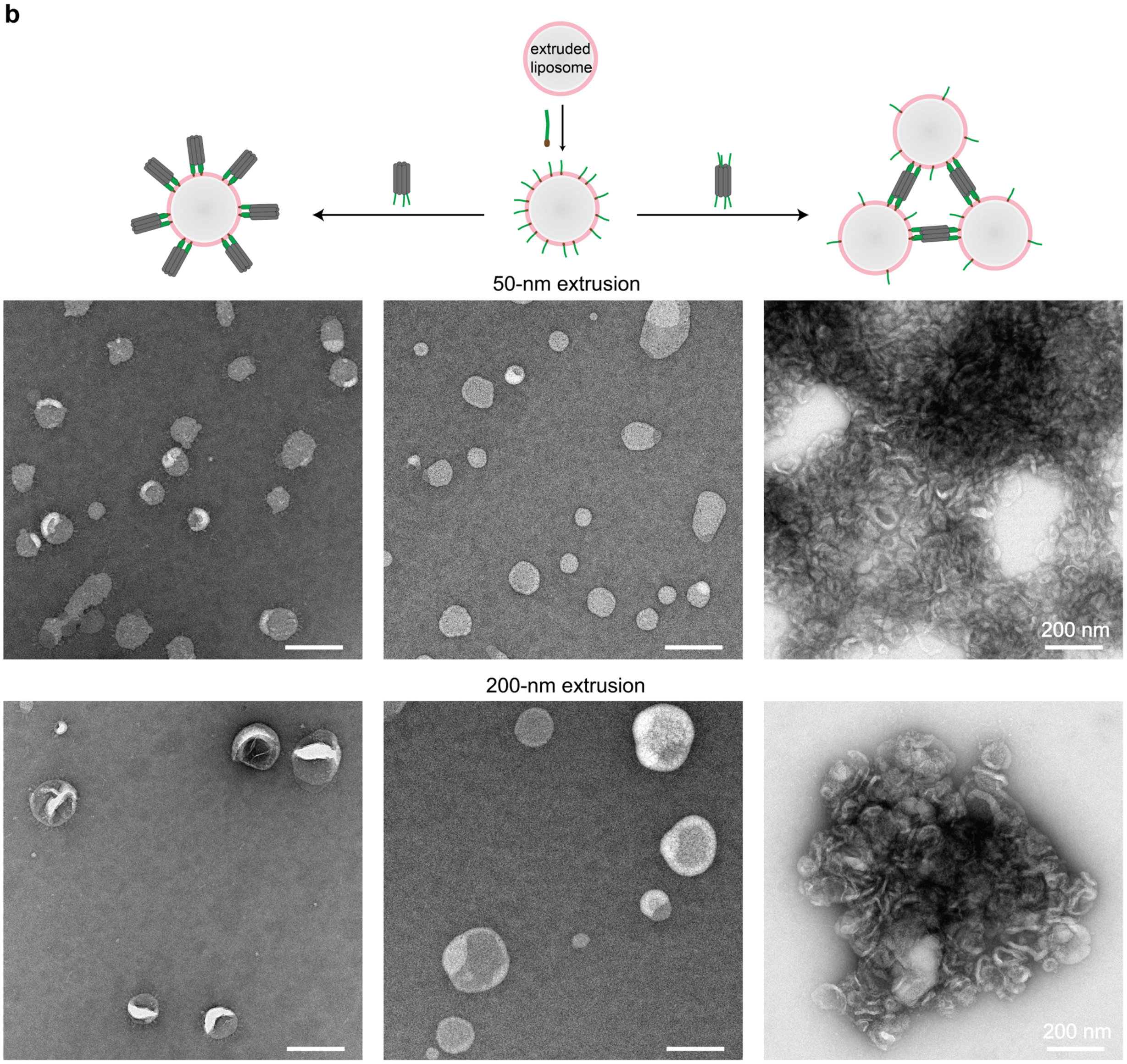

Supplementary Figure 21. Coating and clustering extruded liposomes. **a.** Diameter of liposomes prepared by either dialysis (for detergent removal) or extrusion (through filters with pores of 50 or 200 nm) measured in negative-stain TEM images. N = 58, 56, 30 for each sample from left to right. Source data are provided as a Source Data file. Mean diameter values with

standard deviations are listed in the table (left) and are plotted in the bar graph with error bar (right). In general, extruded liposomes are larger and less homogeneous in size than dialyzed liposomes. **b.** Representative negative-stain TEM images show successful coating (left) or clustering (right) of two different-sized extruded liposomes (central) by 6HB DNA tiles. Scale bars: 200 nm.

Experimental protocol for liposome extrusion: Lipid mixture of 65% DOPC, 15% DOPE, and 20% DOPS (1.5 μmol total lipid) was nitrogen-dried and vacuum-dried in a glass tube before rehydration with 500 μl buffer (25 mM HEPES, 100 mM KCl, pH 7.4) by vortexing for 10 minutes. The solution was transferred into a 1.5 ml centrifuge tube and subjected to five freeze-thaw cycles between a liquid nitrogen bath (-196 °C) and a 37 °C water bath. Finally, the solution was sequentially extruded through polycarbonate filters with pore sizes of 50 or 200 nm, using a Mini Extruder (>40 passes each) at room temperature.

Supplementary Reference

1. Tian, C. et al. Approaching the Limit: Can One DNA Strand Assemble into Defined Nanostructures? *Langmuir* **30**, 5859-5862 (2014).
2. Denisov, I. G., Baas, B. J., Grinkova, Y. V. & Sligar, S. G. Cooperativity in Cytochrome P450 3A4: LINKAGES IN SUBSTRATE BINDING, SPIN STATE, UNCOUPLING, AND PRODUCT FORMATION*. *J. Biol. Chem.* **282**, 7066-7076 (2007).
3. Zhang, Z. & Chapman, E. R. Programmable Nanodisc Patterning by DNA Origami. *Nano Lett.* **20**, 6032-6037 (2020).
4. Weber, T. et al. SNAREpins: Minimal Machinery for Membrane Fusion. *Cell* **92**, 759-772 (1998).
5. Parlati, F. et al. Rapid and efficient fusion of phospholipid vesicles by the α-helical core of a SNARE complex in the absence of an N-terminal regulatory domain. *Proc. Natl. Acad. Sci. U.S.A.* **96**, 12565-12570 (1999).
6. Weber, T. et al. Snarepins Are Functionally Resistant to Disruption by Nsf and αSNAP. *J. Cell Biol.* **149**, 1063-1072 (2000).
7. Scott, B. L. et al. Liposome Fusion Assay to Monitor Intracellular Membrane Fusion Machines. *Methods Enzymol.* **372**, 274-300 (2003).
8. Douglas, S. M. et al. Rapid prototyping of 3D DNA-origami shapes with caDNAno. *Nucleic Acids Res.* **37**, 5001-5006 (2009).
9. Mathieu, F. et al. Six-Helix Bundles Designed from DNA. *Nano Lett.* **5**, 661-665 (2005).
10. Wang, P. et al. Programming Self-Assembly of DNA Origami Honeycomb Two-Dimensional Lattices and Plasmonic Metamaterials. *J. Am. Chem. Soc.* **138**, 7733-7740 (2016).
11. Douglas, S. M., Chou, J. J. & Shih, W. M. DNA-nanotube-induced alignment of membrane proteins for NMR structure determination. *Proc. Natl. Acad. Sci. U.S.A.* **104**, 6644-6648 (2007).
12. Douglas, S. M. et al. Self-assembly of DNA into nanoscale three-dimensional shapes. *Nature* **459**, 414-418 (2009).
13. Lin, C. et al. Purification of DNA-origami nanostructures by rate-zonal centrifugation. *Nucleic Acids Res.* **41**, e40 (2013).
14. Yang, Y. et al. Self-assembly of size-controlled liposomes on DNA nanotemplates. *Nat. Chem.* **8**, 476-483 (2016).
15. Mastronarde, D. N. Automated electron microscope tomography using robust prediction of specimen movements. *J. Struct. Biol.* **152**, 36-51 (2005).
16. Zheng, S. Q. et al. MotionCor2: anisotropic correction of beam-induced motion for improved cryo-electron microscopy. *Nat. Methods* **14**, 331-332 (2017).
17. Mastronarde, D. N. & Held, S. R. Automated tilt series alignment and tomographic reconstruction in IMOD. *J. Struct. Biol.* **197**, 102-113 (2017).
18. Morzy, D. et al. Cations Regulate Membrane Attachment and Functionality of DNA Nanostructures. *J. Am. Chem. Soc.* **143**, 7358-7367 (2021).

19. Beales, P. A. & Vanderlick, T. K. DNA as Membrane-Bound Ligand-Receptor Pairs: Duplex Stability Is Tuned by Intermembrane Forces. *Biophys. J.* **96**, 1554-1565 (2009).
