# Supplementary material for: Functionalization and higher-order organization of liposomes with DNA nanostructures
Source: Nat Commun. 2023 Aug 29;14:5256. doi: 10.1038/s41467-023-41013-2 (PMC10465589; doi:10.1038/s41467-023-41013-2)
Supplement: Supplementary file 4 — Description of Additional Supplementary files [file 41467_2023_41013_MOESM4_ESM.pdf]

Description of Additional Supplementary files

File name: Supplementary Movie 1
Description: 7 cryo tomo slice of tile-mediated liposome cluster.
